# Downregulation of hnRNPA1 inhibits hepatocellular carcinoma cell progression by modulating alternative splicing of ZNF207 exon 9

**DOI:** 10.3389/fonc.2024.1517459

**Published:** 2025-01-06

**Authors:** Qi Ouyang, Wenhui He, Yiping Guo, Lin Li, Ying Mao, Xiang Li, Shuanglin Xiang, Xiang Hu, Jun He

**Affiliations:** ^1^ Hunan Provincial Key Laboratory of Regional Hereditary Birth Defects Prevention and Control, Changsha Hospital for Maternal & Child Health Care Affiliated to Hunan Normal University, Hunan Normal University, Changsha, China; ^2^ State Key Laboratory of Developmental Biology of Freshwater Fish, College of Life Sciences, Hunan Normal University, Changsha, China

**Keywords:** liver cancer, zinc finger protein 207, alternative splice, gene regulation, PI3K/AKT/mTOR

## Abstract

**Introduction:**

Hepatocellular carcinoma (HCC) is the most prevalent liver cancer and a leading cause of cancer-related deaths worldwide. Heterogeneous nuclear ribonucleoprotein A1 (hnRNPA1) plays a critical role in RNA metabolism, including alternative splicing, which is linked to cancer progression. Our study investigated the role of *hnRNPA1* in HCC and its potential as a therapeutic target.

**Methods:**

We analyzed *hnRNPA1* expression in HCC tissues compared to non-tumor tissues using RNA-seq and immunohistochemistry. *hnRNPA1* was knocked down in Hep G2 cells to assess its impact on cell proliferation, migration, and apoptosis using scratch assays, flow cytometry, qPCR, and Western blot. We also explored the interaction between *hnRNPA1* and ZNF207, as well as its splicing effects and downstream signaling pathways by RIP assay, bioinformatics, qPCR and Western blot.

**Results:**

*hnRNPA1* was significantly upregulated in HCC tissues compared to normal tissues, correlating with poor patient survival. *hnRNPA1* knockdown reduced Hep G2 cell proliferation and migration while increasing apoptosis. We identified that *hnRNPA1* bound to ZNF207 and regulated its exon 9 skipping, influencing ZNF207 splicing and the PI3K/Akt/mTOR pathway, key regulators of cell growth and survival.

**Conclusion:**

Our findings demonstrate that *hnRNPA1* promotes HCC progression by regulating ZNF207 splicing and the PI3K/Akt/mTOR pathway. hnRNPA1-ZNF207 interaction represents a potential therapeutic target for HCC, providing insights into the molecular mechanisms underlying HCC progression.

## Graphical Abstract

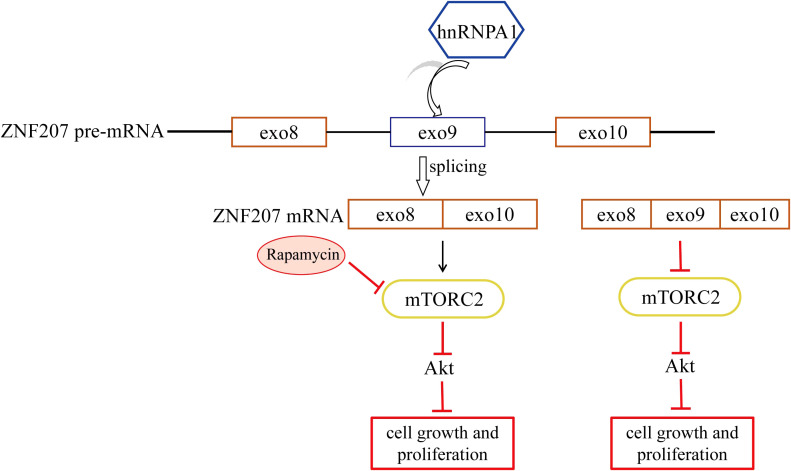

## 1 Introduction

Liver cancer is a significant global health concern, ranking as the sixth most commonly diagnosed cancer and the third leading cause of cancer-related deaths worldwide (1). In 2020 alone, it accounted for over 900,000 new cases and more than 830,000 fatalities. Hepatocellular carcinoma (HCC), the most prevalent form, constitutes over 80% of all liver cancer cases. HCC is notorious for its high propensity for metastasis and postoperative recurrence, which significantly challenge treatment outcomes and patient survival (2). Metastasis often occurs when cancer cells from the primary liver tumor spread to distant organs such as the lungs, bones, or brain (3). This process involves complex interactions between cancer cells and the surrounding microenvironment, enabling the cells to invade adjacent tissues, enter the bloodstream, and establish secondary tumors (4). Postoperative recurrence, where the cancer returns after surgical removal of the primary liver tumor, is another major hurdle. It can be due to microscopic cancerous cells that remain undetected during surgery, microvascular invasion or the presence of occult metastasis (5, 6). These factors contribute to the aggressive nature of HCC and emphasize the need for advanced treatment strategies and careful post-surgery monitoring to improve patient outcomes. In recent years, plant-derived natural products and their metabolites have gained attention for their potential to inhibit HCC. Compounds such as polyphenols and flavonoids have demonstrated antitumor effects by targeting key pathways involved in HCC progression (7, 8).

RNA-binding proteins are critical *trans*-factors that specifically bind to *cis* elements in mRNAs, exerting regulatory control over mRNA stability and translation (9, 10). An essential process in RNA regulation is alternative splicing (AS), which generates different mRNA splicing isomers from pre-mRNA through diverse splicing methods (11). Microarray analyses reveal that over 95% of human genes are subject to AS, leading to the generation of various protein isoforms (12). The process includes several types of AS, including exon skipping, mutually exclusive exons, alternative 3′ and 5′ splice site selection, and intron retention (13). Growing evidence has shown that cancer cells exploit this mechanism to enhance their growth and metastatic potential by manipulating splicing patterns. Among the key players in this regulation are heterogeneous nuclear ribonucleoproteins (hnRNPs). These proteins are crucial in regulating selective splicing of pre-mRNAs and stabilizing mRNA translocations, thus influencing the gene expression landscape in cancer cells (14, 15). hnRNP proteins regulate all levels of expression of apoptotic genes, including transcription initiation and elongation, alternative splicing, mRNA stability, translation, and protein degradation (16). For example, splicing to the Bcl-xS 5′ splice site was also enforced by heterogeneous nuclear ribonucleoprotein (hnRNP) F/H proteins and by Sam68 in cooperation with hnRNPA1. Overall several reports suggest hnRNPA1 regulates the binding ability of genes with RNA through post-transcriptional modifications such as phosphorylation and ubiquitination, thereby affecting their roles in cell proliferation and apoptosis (17–19).

Zinc finger protein 207 (ZNF207) is part of the zinc finger family of transcription factors, which plays a crucial role in binding to specific DNA sequences to regulate gene expression (20). Recent study has identified ZNF207 as a putative immunosuppressive target in HCC (21), which demonstrated that ZNF207 protein levels are significantly elevated in HCC tissues, and its expression is associated with several clinical parameters such as cirrhosis, nodule number, tumor capsule presence, vascular invasion, and TNM staging (tumor, Node, Metastasis staging system), all of which are critical factors in the progression and prognosis of HCC (22). The role of ZNF207 in cancer, particularly in HCC, underscores its importance in the field of oncology, aligning with the broader research on zinc finger proteins in cancer. The connection between high ZNF207 expression and key pathological features of HCC suggests that ZNF207 could serve as a biomarker for HCC severity and progression. Furthermore, its involvement in these crucial aspects of HCC makes it a compelling candidate for precision therapy, targeting specific molecular pathways to improve treatment outcomes (23). Despite these insights, the exact mechanisms through which ZNF207 contributes to HCC progression remain unclear.

The phosphoinositide 3-kinase/protein kinase B/mammalian target of rapamycin (PI3K/Akt/mTOR) is a well-established signaling cascade critical for cell proliferation and differentiation (24). This pathway is initiated when cell surface receptors respond to external stimuli, activating PI3K. Subsequently, PI3K catalyzes the conversion of phosphatidylinositol 4,5-bisphosphate into phosphatidylinositol-3,4,5-trisphosphate, which in turn activates Akt. And then Akt stimulates mTOR complex 1 (mTORC1), enhancing the cellular synthesis of proteins, nucleotides, and lipids, thus promoting cell proliferation. Dysregulation of this pathway can lead to excessive cell proliferation, commonly associated with disease states, including cancer (25). Rapamycin, an allosteric inhibitor of mTOR, was initially approved as an immunosuppressant but has since garnered attention for its potential as an anticancer agent (26). Despite the broad implications of mTOR inhibitors in cancer therapy, the link between aberrant alternative splicing, a process that can influence cancer progression by generating diverse protein isoforms, and mTOR activation in HCC remains unexplored.

In light of the intricate mechanisms that govern HCC progression and the significant role of RNA-binding proteins in regulating gene expression, this study focuses on hnRNPA1 and its potential impact through the modulation of AS. Particularly, we examine the splicing of exon 9 in the ZNF207 gene, hypothesizing that hnRNPA1’s interaction with this process could crucially influence the PI3K/Akt/mTOR signaling pathway, a key regulator of cellular behaviors associated with cancer aggressiveness such as proliferation and migration. By delineating how hnRNPA1 affects this pathway via ZNF207, we aim to uncover novel therapeutic targets and provide a more profound understanding of the molecular underpinnings of HCC. This could pave the way for developing strategies that inhibit HCC progression by targeting specific components of its genetic regulation.

## 2 Materials and methods

### 2.1 Bioinformatics analysis

Gene expression data were sourced from GSM2343352 and GSM2343354. The analysis and visualization of gene expression were conducted using the GEPIA (Gene Expression Profiling Interactive Analysis) online tool (http://gepia.cancer-pku.cn) and the ggplot2 package in R (https://www.r-project.org/). Survival analysis was performed and visualized using the “survival” package (https://cistrome.shinyapps.io/timer/). Raw data were compared and quantified using RSEM 1.3.3 and Bowtie2 software (27, 28). Alternative splicing was analyzed using rMATS 4.1.2 software, with visualization of these results facilitated by rmats2sashimiplot (29). Differentially expressed genes (DEGs) was identified using the “DESeq2” package (https://bioconductor.org/packages/release/bioc/html/DESeq2.html), and Gene Ontology (GO) functional enrichment and Kyoto Encyclopedia of Genes and Genomes (KEGG) pathway enrichment analyses were conducted using the “cluster Profiler” package (https://bioconductor.org/packages/release/bioc/html/clusterProfiler.html).

### 2.2 Cell culture and cell viability assay

Hep G2 cells and SMMC 7721 were obtained from the Cell Bank of the Chinese Academy of Sciences and cultured according to the study’s protocol. These cells were maintained in DMEM enriched with 10% heat-inactivated FBS, penicillin (100 units/mL), and streptomycin (100 μg/mL) in a 37°C incubator with a humidified atmosphere of 5% CO_2_. Cell viability was assessed using the MTT assay. For this assay, cells were seeded in 96-well plates and treated with the appropriate reagents. After treatment, 100 μL of MTT solution (5 mg/ml) was added to each well, and the plates were incubated for 4 h at 37°C. Subsequently, 100 μL of DMSO (Sangong, Shanghai) was used to dissolve the formazan crystals formed by the cells. The optical density was then measured at 492 nm using a PL-9602 Enzyme Labeling Instrument (Perlong Medical, Beijing, China). Cell viability was calculated as a percentage relative to the control.

### 2.3 Construction of mutant plasmid

The *hnRNPA1* overexpression plasmid hnRNPA1 (NM_002136) pcDNA3.1-3xFlag-C was purchased from Youbio (Changsha, Hunan). For the construction of the pCMV-Myc-ZNF207 mutant plasmid, primers ZNF207-Bal I, ZNF207-Kpn I, and ZNF207-OE-F/R were designed (**Supplementary Table S1**). Overlapping PCR was performed using ZNF207-Bal I and ZNF207-OE-R, as well as ZNF207-Kpn I and ZNF207-OE-F, under the following conditions: 95°C for 3 min, followed by 29 cycles of 95°C for 30 s, 58°C for 30 s, and 72°C for 10 s, with a final extension at 72°C for 5 min. The PCR products were then combined and used as a template for a subsequent round of PCR with ZNF207-Bal I-F and ZNF207-Kpn I-R. The pCMV-Myc plasmid served as the template for these PCR amplifications. Next, 20 μL of the PCR product was mixed with 1 μL of Bal I and 1 μL of Kpn I (Thermo Fisher Scientific, USA), and the mixture was incubated at 37°C for 14 h. Homologous recombination was then carried out at the mutant sites of the overlapping products using reagents from T4 DNA Ligase (Thermo Fisher Scientific, USA). Finally, 5 μL of the recombinant plasmid was transformed into Top10 competent cells and cultured on plates containing ampicillin overnight. The correctly constructed plasmid was confirmed through nucleic acid sequencing.

### 2.4 Immunohistochemical (lHC) staining

We collected tumor samples from patients. Briefly, the hnRNPA1 antibodies (1:100) were applied to stain each tissue sample. Subsequently, the indicated HRP-conjugated secondary antibody was used in incubating the slice at 37 °C for 45 min. We then used diaminobenzidine (DAB)solution (Immunoway, China) to stain the slice, and we counterstained the nuclei with Harris’hematoxylin. The percentage of specifically positive staining of tumor cells was classified with the following grades: 0 (<5%), 1 (6%-25%), 2 (26%-50%), 3 (51%-75%), and 4 (>75%). The final score was expressed by multiplying the staining intensity and the percentage of specifically positive staining tumor cells.

### 2.5 RNA extraction and RT-qPCR

RNAiso easy (Takara, Japan) was used to extract total RNA according to the manufacturer’ instructions. cDNA was synthesized from 1 μg of total RNA using NovoScripts plus All-in-one 1st Strand cDNA Synthesis Super Mix (E401, Novoprotein, Suzhou, China), following the manufacturer’s instructions. Real-time RT-PCR analysis was performed on a CFX Connect PCR detection system (Bio-Rad, Germany) using NovoStart Universal Fast SYBR qPCR Super Mix (E047, Novoprotein, Suzhou, China), according to the manufacturer’s instructions. The reaction was performed on CFX Conect (Bio-Rad, Singapore) with conditions: 95°C for 2 min, 38 cycles of 95°C for 5 s, and 60°C for 15s. The mRNA expression (folds) was calculated using the 2^−ΔΔCt^ method.

### 2.6 Cell transfection

The siRNAs of *hnRNPA1* and control were purchased from Jikai Gene (Suzhou, China). Sequences are detailed in **Supplementary Table S1**. Cells grown in 6-well plates were transfected with a final concentration of 75 pmol. Lipofectamine 2000 (Invitrogen) was used for all transfection assays according to the instructions. The efficiency of all transfections was examined by RT-qPCR. Relative sequences are listed in **Supplementary Table S1**.

### 2.7 Flow cytometry analysis

SMMC 7721 or Hep G2 were seeded onto a 6-well plate at a density of 5 × 10^5^ per well 36 h before transfection with si-*hnRNPA1*. To analyze apoptosis transfected si-*hnRNPA1*, cells were collected and double-stained with annexin V and propidium iodide using the Annexin V-PI apoptosis detect in Kit (Vazyme, Nanjing, China), following the manufacturer’s instructions. The flow data were selected using BD FACSDiva v.7 and analyzed using FlowJo v.7.6. The gating strategies are shown in **Supplementary Figure S5**.

### 2.8 Cell clone formation and cell wound healing assay

For the cell clone formation assay, 1,000 transfected cells were seeded in 6-well plates. After four days, the cells were fixed with 4% paraformaldehyde (PFA) for 30 min. Following fixation, cells were rinsed with tap water and stained with a 0.1% crystal violet solution for 1 h. The plates were rinsed again, and photographs were taken for documentation.

For the wound healing assay, cells were plated in 6-well plates and allowed to grow until they reached 100% confluence. A scratch was then made on the plate using a 10 μL pipette tip, and the cells were subsequently cultured in serum-free DMEM medium. For each sample, three random areas were selected and observed under 10 x 10 magnification at 0 and 24 h, respectively. The relative migration distance of the cells was measured to assess their migration capability.

### 2.9 Western blot analysis

Treated cells were lysed with RIPA lysis buffer supplemented with a protease inhibitor and phosphatase inhibitor (ABclonal, China) for 30 min on ice. The protein samples were collected after centrifugation at 12,000 rpm at 4°C for 10 min. Equal amounts of protein were loaded and separated on 6%~15% SDS-PAGE gels and transferred onto polyvinylidene difluoride (PVDF) membranes (Merck Millipore Ltd. IPVH00010, Darmstadt, Germany). The membranes were blocked with 5% milk blocking solution at room temperature for 1-2 h, washed in TBST buffer, and incubated overnight at 4°C with primary antibodies of p-mTOR, mTOR, p-PI3K and PI3K (1:1000, ZEN-BIOSCIENCE, China), p-Akt, Akt, hnRNPA1 (1:1000, Immunoway, China), β-actin, β-tubulin and GAPDH (1:2000, ABclonal, China), Bax, Bcl2, Caspase 3 and cleaved Caspase3 (1:1000, HuaBio, China). After washing with TBST 3 times, the membranes were incubated with secondary antibodies conjugated with horseradish peroxidase (HRP) (1:10000, Abbkine, China) at room temperature for 1 h. The membrane blots were detected by using an enhanced chemiluminescence (ECL) kit (NCM Biotech, China). All gray analyses for protein blots were performed with ImageJ software.

### 2.10 TBE vertical gel electrophoresis

Gel electrophoresis at 5% concentration was performed using the following components: 6.5 mL of ddH_2_O, 1.25 mL of 40% acrylamide, 2 mL of 5 X TBE, 100 μL of 10% ammonium persulfate (AP, Sangon, China), and 8 μL of TEMED. After the gel solidified, DNA samples from the transfected cells were loaded, and the electrophoresis was conducted at 125 V and 125 mA for 60 minutes. Subsequently, the gel was immersed in 4S Red stain (Sangon, China) and allowed to develop under a UV lamp for one hour.

### 2.11 RNA immunoprecipitation assays

Cells were lysed by multimeric lysate contained with 1 M KCl, 50 mM MgCl_2_, 100 mM HEPEs-NaOH (PH 7), 5% NP-40, 1mM DTT, 200 units/mL RNase Inhibitor (Sangon Biotech, B600478), Protease Inhibitor Cocktail (MedChemExpress, HY-K0010). Then, protein A/G magnetic beads (Selleck, B23201) preincubated with IgG (negative control) or antibody specific for HNRNPA1 (Abclonal, A11564) were incubated with lysates at 4°C overnight. After washing five times, Eluted RNAs were purified by Proteinase K (Sangon Biotech, A610451) The enrichment of ZNF207 was detected by qRT-PCR.

### 2.12 Statistical analysis

Data are presented as means ± standard error (SD). Comparisons among groups were performed using one-way ANOVA followed by Dunnett’s test using GraphPad Prism 9.0 with different letters representing different significances: a: *p*< 0.05, b: *p*< 0.01, c: *p*< 0.005, d: *p*< 0.001 (*n* = 3).

## 3 Results

### 3.1 Expression patterns of *hnRNPA1* in HCC

**Figure 1:**
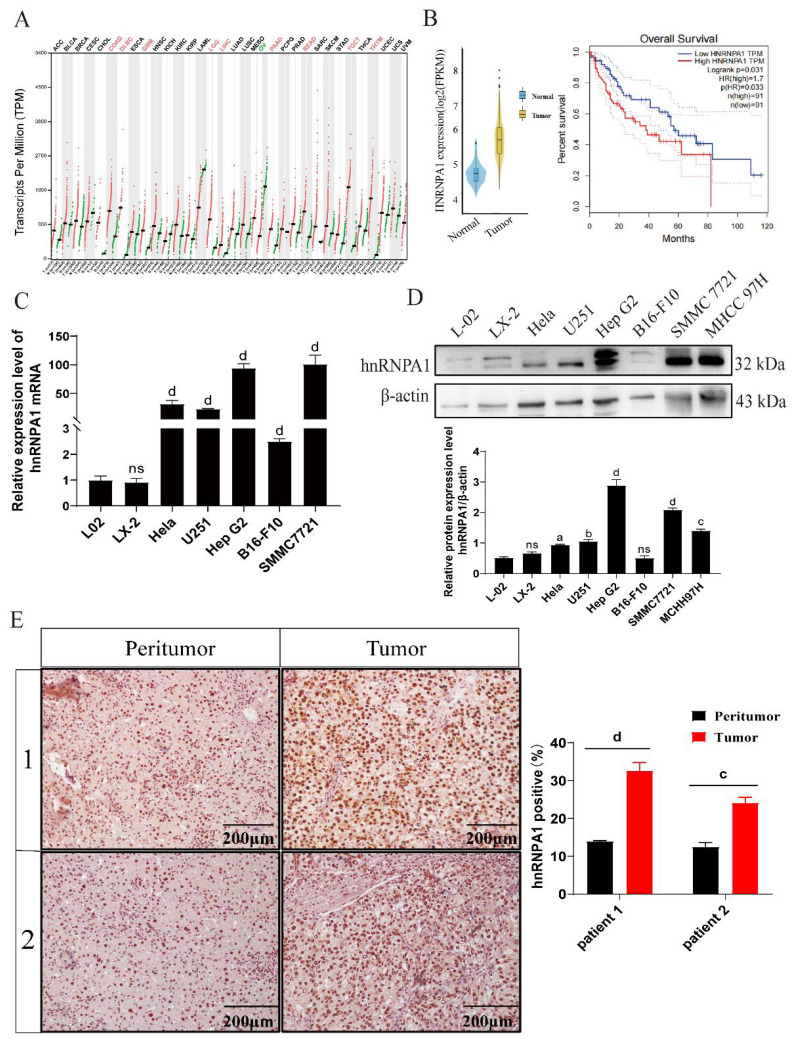
The expression levels of hnRNPA1 in HCC. **(A)** *hnRNPA1* expression profile across all tumor samples and paired normal tissues. Red letters indicate high expression of *hnRNPA1* in this tissue. Green letters indicate low expression of *hnRNPA1* in this tissue. **(B)** Kaplan-Meier survival curves of HCC patients. **(C)** Relative mRNA expression of *hnRNPA1* in different cells by quantitative real-time PCR (*n* = 3). Data are normalized to the *Gapdh* mRNA. Different letters indicate statistical significance (*ns*: no significance, d: *p* < 0.001). **(D)** Western blot analysis of hnRNPA1 in different cells. β-actin was set as the control group. Original images are presented in **Supplementary Figure S1**. *ns*: no significance, a: *p* < 0.05, b: *p* < 0.01, c: *p* < 0.005, d: *p* < 0.001. **(E)** Representative immunohistochemistry images for hnRNPA1 protein expression in peritumor and HCC tumor tissues. c: *p* < 0.005, d: *p* < 0.001. Scale bar, 200 μm.

Using bioinformation tool, we analyzed gene expression profiles from 374 cancer tissue samples and 50 normal tissue samples extracted from The Cancer Genome Atlas (TCGA) database. Our study identified a significant elevation in *hnRNPA1* expression in different cancers such as colorectal adenocarcinoma (COAD), diffuse large B-cell lymphoma (DLBC), and liver hepatocellular carcinoma (LIHC) (**Figure 1A**). Kaplan-Meier survival curves further indicated a negative correlation between *hnRNPA1* expression and survival rates in HCC patients (**Figure 1B**). Specifically, the mRNA expression level of *hnRNPA1* was increased in the HCC cell (SMMC 7721 and Hep G2) compared with normal liver cells (L02 and LX-2), also the mRNA expression level of *hnRNPA1* was increased in other cancer cells (U251, Hela and B16-F10) (*p* < 0.001) (**Figure 1C**). The result of western blot analysis confirmed that hnRNPA1 expression was predominantly elevated in HCC cells (SMMC 7721, MCHH 97H and Hep G2) in comparison to non-HCC cells (Hela, B16-F10 and U251) and normal liver cells (L-02 and LX-2) (*p* < 0.05) (**Figure 1D**; **Supplementary Figure S1**). The size difference in hnRNPA1 protein originates from the alternative splicing in the C-terminus. Immunohistochemistry performed on HCC patient tissues validated the significant elevation of hnRNPA1 in tumor tissues compared to adjacent non-tumor tissues (*p* < 0.001) (**Figure 1E**).

### 3.2 Knockdown of *hnRNPA1* inhibited HCC cells proliferation and migration

To elucidate the role of *hnRNPA1* in facilitating the progression of HCC and its underlying molecular mechanisms, we engineered siRNAs targeting *hnRNPA1* specifically (**Supplementary Table S1**, **Supplementary Figure S2A**). MTT and clonogenic assays revealed that silencing *hnRNPA1* significantly reduced the proliferation of Hep G2 cells (*p* < 0.05), (**Supplementary Figures S2B, C**). Additionally, wound healing assays demonstrated that *hnRNPA1* knockdown markedly decreased the migration of Hep G2 cells (*p* < 0.05, **Supplementary Figure S2D**). Previous research indicates that *hnRNPA1* suppresses pro-apoptotic proteins, thereby influencing tumor growth (30). Western blot analysis confirmed that *hnRNPA1* knockdown reduced Bcl2 levels and enhanced the expression of the pro-apoptotic protein Bax and caspase-3 activation (**Supplementary Figure S2E**).

**Figure 2:**
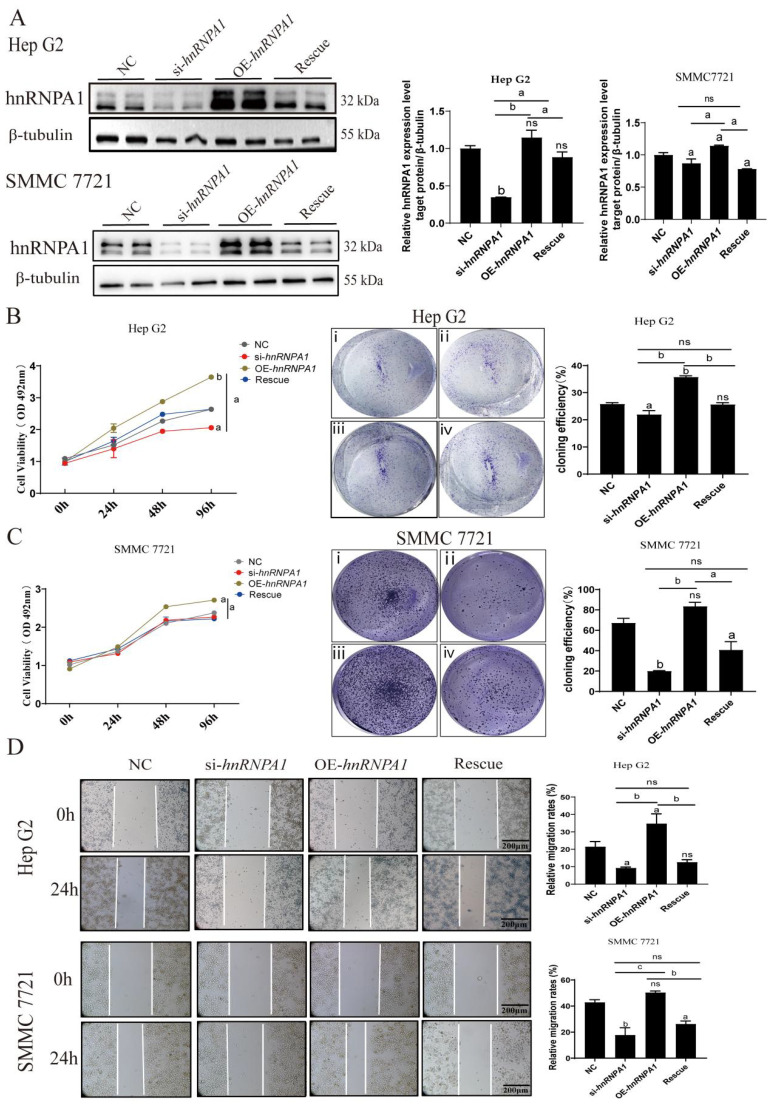
Knockdown of *hnRNPA1* inhibits HCC cells proliferation and migration. **(A)** Western blot analysis of hnRNPA1 in Hep G2 and 7721. β-tubulin was set as the control group. Original blots are presented in **Supplementary Figure S3A**. **(B)** MTT assay showed that *hnRNPA1* knockout inhibited cells proliferation. *ns*, no significance, a: *p* < 0.05, b: *p* < 0.01. **(C)** Cell clone formation assay showed that *hnRNPA1* knockout inhibited cells proliferation. *hnRNPA1* overexpression promoted the proliferation of Hep G2. i, NC, ii, si-*hnRNPA1*, iii, OE-*hnRNPA1*, iv, Rescue. **(D)** Wound healing assay showed that *hnRNPA1* knockout inhibited cells migration within 24 h. *hnRNPA1* overexpression promoted cells migration. *ns*, no significance, a: *p* < 0.05, b: *p* < 0.01, c: *p* < 0.005. Scale bar, 200 μm.

Subsequently, we assessed *hnRNPA1* expression in Hep G2 and SMMC 7721 cells (**Figure 2A**; **Supplementary Figure S3A**). MTT and clonogenic assays showed that overexpressing *hnRNPA1* significantly increased cell proliferation in both Hep G2 and 7721 cells, significance is only evaluated between knockdown and overexpressing cells (**Figures 2B, C**). The above results showed that cell proliferation was no significant difference within 24h. The result of wound healing assays showed that overexpression of *hnRNPA1* led to a significantly higher migration rate compared to the control within 24h (**Figure 2D**). CCND1 (Cyclin D1), MPP2 (Membrane palmitoylated protein 2), Bax, Bcl2, HIF1A and VEGFA are transcriptionally regulated molecules that are related to cell proliferation, apoptosis and cancer cell invasion and metastasis processes. The results of qPCR showed that knockdown-*hnRNPA1* dramatically lowered mRNA levels of these molecules in Hep G2 (**Supplementary Figure S3B**).

### 3.3 Knockdown of *hnRNPA1* functional enrichment analysis

**Figure 3:**
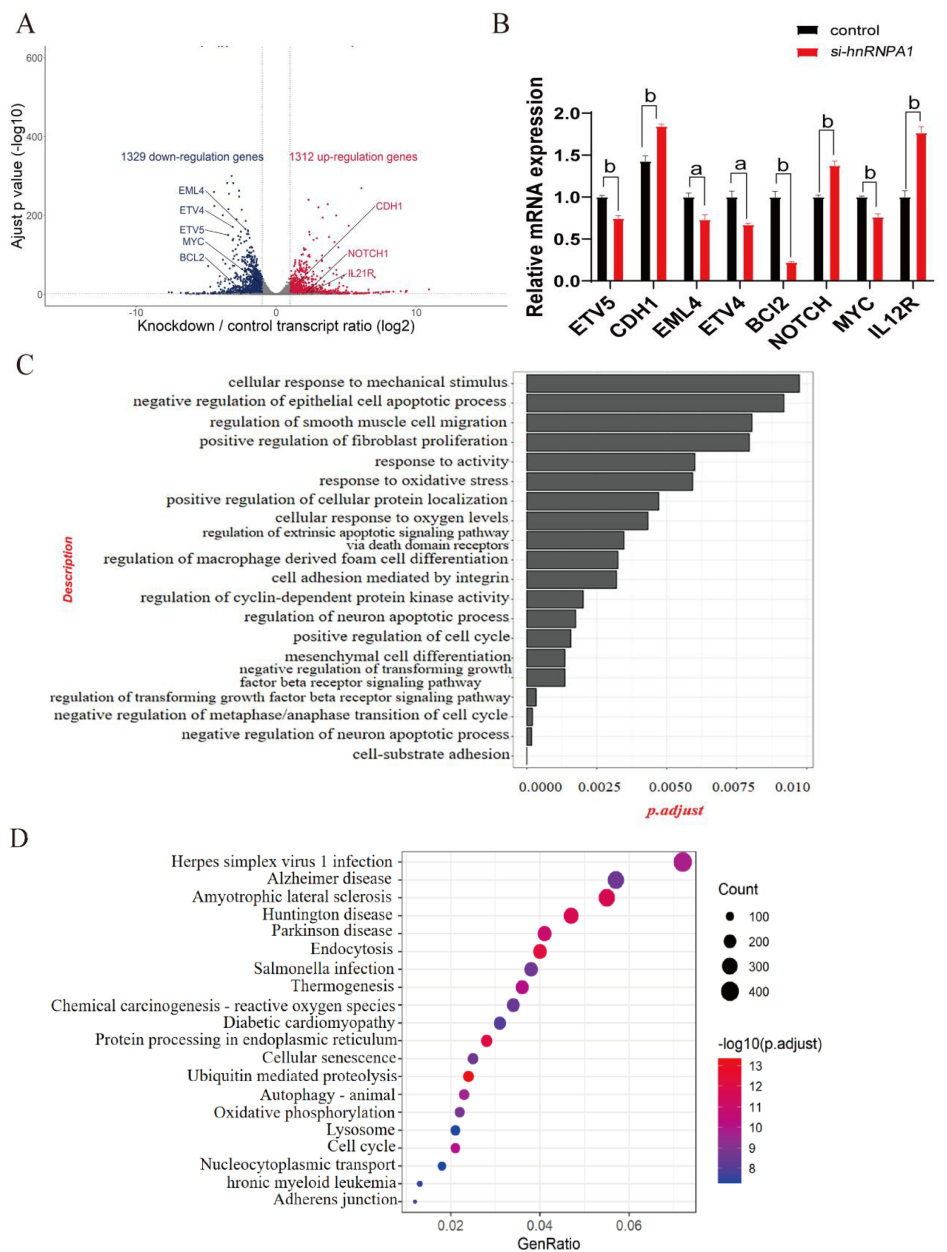
Differentially expressed genes (DEGs) identification and functional enrichment analysis of *hnRNPA1*-knockdown cells. **(A)** Volcano plot of DEGs identified in the comparison after *hnRNPA1* knockdown. **(B)** Relative mRNA expression of genes in Hep G2 cells by quantitative real-time PCR (n = 3). Data are normalized to the *Gapdh* mRNA. Different letters indicate statistical significance. a: *p*<0.05, b: *p*<0.01. **(C)** GO enrichment analysis of DEGs after *hnRNPA1* knockdown. **(D)** KEGG analysis of DEGs after *hnRNPA1* knockdown.

To further explore the relationship between *hnRNPA1* and HCC, we performed an in-depth RNA-seq analysis of *hnRNPA1*-knockdown in Hep G2 cells using common data. The results identified 2,641 DEGS from TCGA, GSM2343352 and GSM2343354, consisting of 1,312 up-regulated genes and t. 1,329 down-regulated genes. Changes in down-regulated genes were ETV4 (ETS Variant Transcription Factor 4) and ETV5 (ETS Variant Transcription Factor 5) are related to transcriptional regulation, EML4 (EMAP like 4), BCL2 and Myc are related to cancer development. And changes in up-regulated genes were CDH1 (E-cadherin), NOTCH1 and IL21R were related to the normal morphology of cells (**Figure 3A**). The results of RT-qPCR were consistent with the results of DEG analysis (**Figure 3B**). GO analysis of the identified DEGs showed that several biological processes (BP) related to tumorigenesis, the apoptosis signaling pathway, and the negative regulation of cell proliferation were all affected after *hnRNPA1* knockdown (**Figure 3C**). KEGG pathway analysis revealed significant differences in the expression of protein processing in endoplasmic reticulum, ubiquitin mediated proteolysis, and endocytosis (**Figure 3D**).

### 3.4 Alternative splicing events mediated by *hnRNPA1* in Hep G2 cells

**Figure 4:**
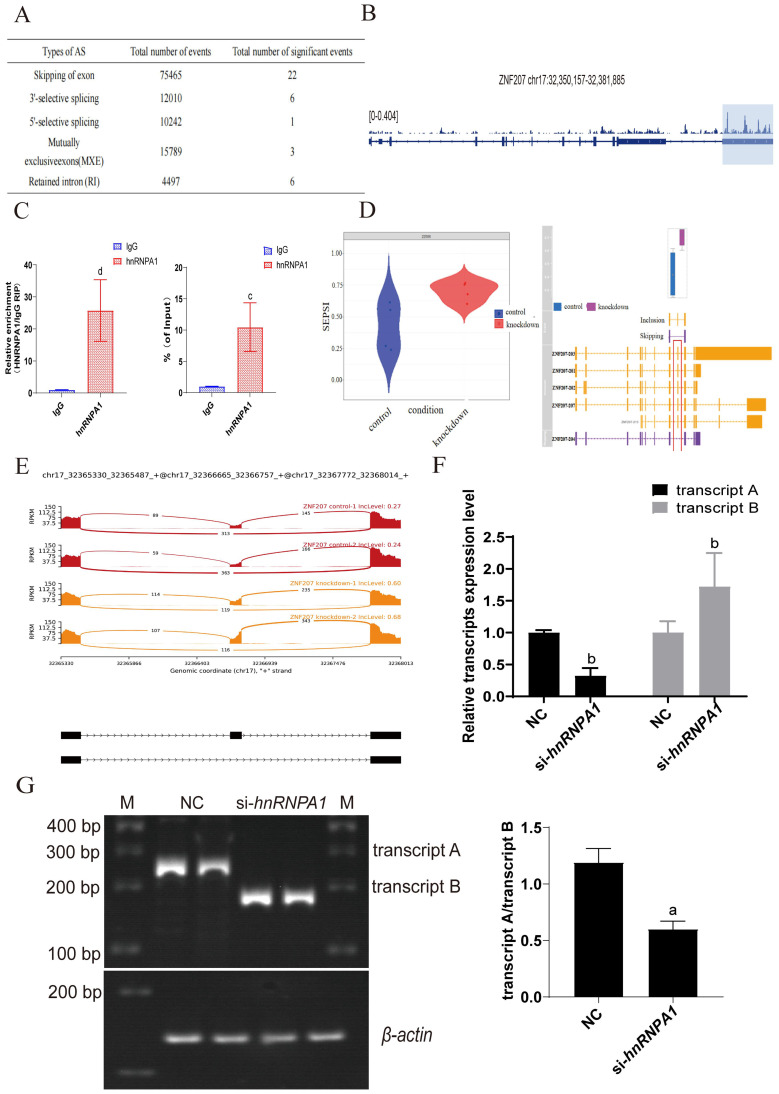
Alternative Splicing Events Mediated by hnRNPA1 in Hep G2 Cells. **(A)** *hnRNPA1* gene regulates the occurrence of AS events. **(B)** Splicing regulation of ZNF207. Tracks indicate eCLIP (reads per million). **(C)** The combination between hnRNPA1 and ZNF207 was analyzed using RIP assay. c: *p* < 0.001, d: *p* < 0.005. **(D)** Expression levels of ZNF207 exon skipping transcripts before and after *hnRNPA1* knockdown. **(E)** Schematic diagram of the ZNF207 isomer. Expression of ZNF207 exon skipping transcripts before and after knockdown *hnRNPA1*. Red areas represented the expression of ZNF207 exon skipping transcript before knockdown *hnRNPA1*. Yellow areas represented the expression of ZNF207 exon skipping transcript after knockdown *hnRNPA1*. **(F)** Relative mRNA expression of transcript A and B in Hep G2 cells by quantitative real-time PCR after *hnRNPA1* knockdown. (n = 3). Data are normalized to the *β-actin* mRNA. Different letters indicate statistical significance. b: *p*<0.01. **(G)** TBE analysis of ZNF207 transcription level in knockdown of *hnRNPA1* and original gels are presented in **Supplementary Figure S4A**. Sample loading has been normalized to the transcriptional level of *β-actin*, a: *p* < 0.05.

It has been reported that *hnRNPA1* plays a crucial role in regulating many cellular AS events. To explore the specific AS events influenced by *hnRNPA1* that affect the proliferation of Hep G2 cells, we processed enhanced ultraviolet cross-linking and immunoprecipitation (eCLIP) and KD-RNA-Seq data from eight samples. We established a genome index with the human genome GRCh38 as the reference, setting the read segment length at 99 for get bam files (31). The files on exon skipping events calculated by rMATS software were analyzed using the maser package in R software and rmats2sashimiplot software, and the occurrence of ZNF207 exon skipping in the control group and the knockdown *hnRNPA1* experimental group was compared to obtaining the regulatory information of *hnRNPA1* on alternative splicing of ZNF207. This analysis identified 75,465 exon skipping (SE) events. Among these, 22 were significantly noteworthy, with ZNF207 displaying the most pronounced change in Delta PSI, showing a decrease of 0.278 (avg reads > 100, FDR > 0.05, Delta PSI = 0.1) (**Figure 4A**). We also noted a substantially increased frequency of exon skipping in ZNF207. Initially, eCLIP data confirmed the binding of *hnRNPA1* to ZNF207 mRNA (**Figure 4B**). RNA immunoprecipitation (RIP) results also revealed that *hnRNPA1* could bind to ZNF207 in Hep G2(**Figure 4C**). Detailed examination indicated that skipping exon 9 in ZNF207 is a specific event, leading to the generation of transcript B (NM00103229.3) (**Figure 4D**). Alternative splicing involves the change of isoforms. Visualization of this AS event revealed elevated IncLevel values for the skipped exon which represented an increased expression of transcript B, which showed by yellow area (**Figure 4E**). The results of TBE blot showed that ratios of transcript A/B were decreased, which indicated the expression of transcript B was higher in Hep G2 (**Figure 4F**; **Supplementary Figure S4A**). Additionally, the result of RT-qPCR showed that the expression of ZNF207 transcript B was increased after *hnRNPA1* knockdown (**Figure 4G**). This suggested that *hnRNPA1* regulate the expression of exon-skipping transcripts of the ZNF207 gene in Hep G2. Skipping of exon 9 in ZNF207 produced transcripts of varying lengths, so the mRNA length changes after splicing.

### 3.5 ZNF207-short but not ZNF207-long promoted cell proliferation and migration

**Figure 5:**
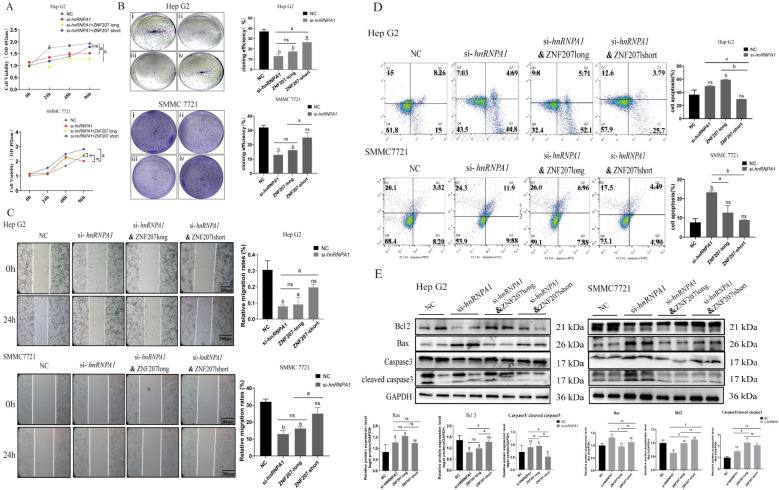
ZNF207-short but not ZNF207-long promotes cells proliferation and migration. **(A)** MTT experiments showed that ZNF207-short, promoted cells proliferation. ns, no significance, a: *p*<0.05, b: *p*<0.01. **(B)** Cell cloning assay showed that ZNF207-short, promoted cells proliferation. i, NC, ii, si-*hnRNPA1*, iii, si-*hnRNPA1*-ZNF 207 long, iv, si-*hnRNPA1-*ZNF 207 short. **(C)** Wound healing assay showed that ZNF207-short, promoted cells migration. a: *p* < 0.05, b: *p* < 0.01. Scale bar, 200 μm. **(D)** Flow cytometry experiments showed that ZNF207-short, inhibited apoptosis in HCC cells. **(E)** Western blot analysis showed that ZNF207-short slowed down the process of apoptosis and original blots are presented in **Supplementary Figure S5B**.

The findings above demonstrated that *hnRNPA1* knockdown impacted AS in Hep G2 cells. To assess the influence of ZNF207 exon 9 splice isoforms on the proliferation and migration of HCC cells, we synthesized mutant plasmids for ZNF207-short and ZNF207-long using overlapping PCR and whole-genome synthesis (**Supplementary Figures S4B, C**). We then evaluated the impact of these mutant plasmids on cell proliferation and migration. Our results indicated that both *hnRNPA1* knockdown and ZNF207-long suppressed HCC cell proliferation whereas ZNF207-short enhanced these processes (**Figures 5A, B**). Wound healing assays further confirmed that knockdown of *hnRNPA1* and ZNF207-long hindered HCC cell migration, while ZNF207-short facilitated it (**Figure 5C**). Additionally, flow cytometry analysis revealed that knockdown of *hnRNPA1* and ZNF207-long increased apoptosis, while ZNF207-short reduced it (**Figure 5D**; **Supplementary Figure S5A**). Western blot analysis showed that knockdown of *hnRNPA1* and ZNF207-long upregulated the pro-apoptotic protein Bax and downregulated the anti-apoptotic protein Bcl2, thereby activating caspase-3 and enhancing apoptosis (**Figure 5E**; **Supplementary Figure S5B**).

### 3.6 mTOR-mediated aberrant alternative splicing affected the PI3K/Akt signaling pathway in HCC cells

Since excessive activation of the PI3K/Akt/mTOR pathway is known to inhibit caspase family proteins, reducing apoptosis, we explored the impact of the ZNF207 splice isoform on this signaling pathway in Hep G2 or SMMC 7721 cells using Western blot analysis. Results indicated that ZNF207-short enhanced the activation of PI3K, leading to its conversion to phosphorylated 3,4,5-phosphatidylinositol triphosphate, which subsequently activated Akt and mTOR, fostering cell proliferation (**Supplementary Figures S6A, B**). Conversely, the knockdown of *hnRNPA1* and ZNF207-long reduced the phosphorylation of PI3K, Akt, and mTOR, with the changes in Akt and mTOR phosphorylation being statistically significant (*p* < 0.05).

**Figure 6:**
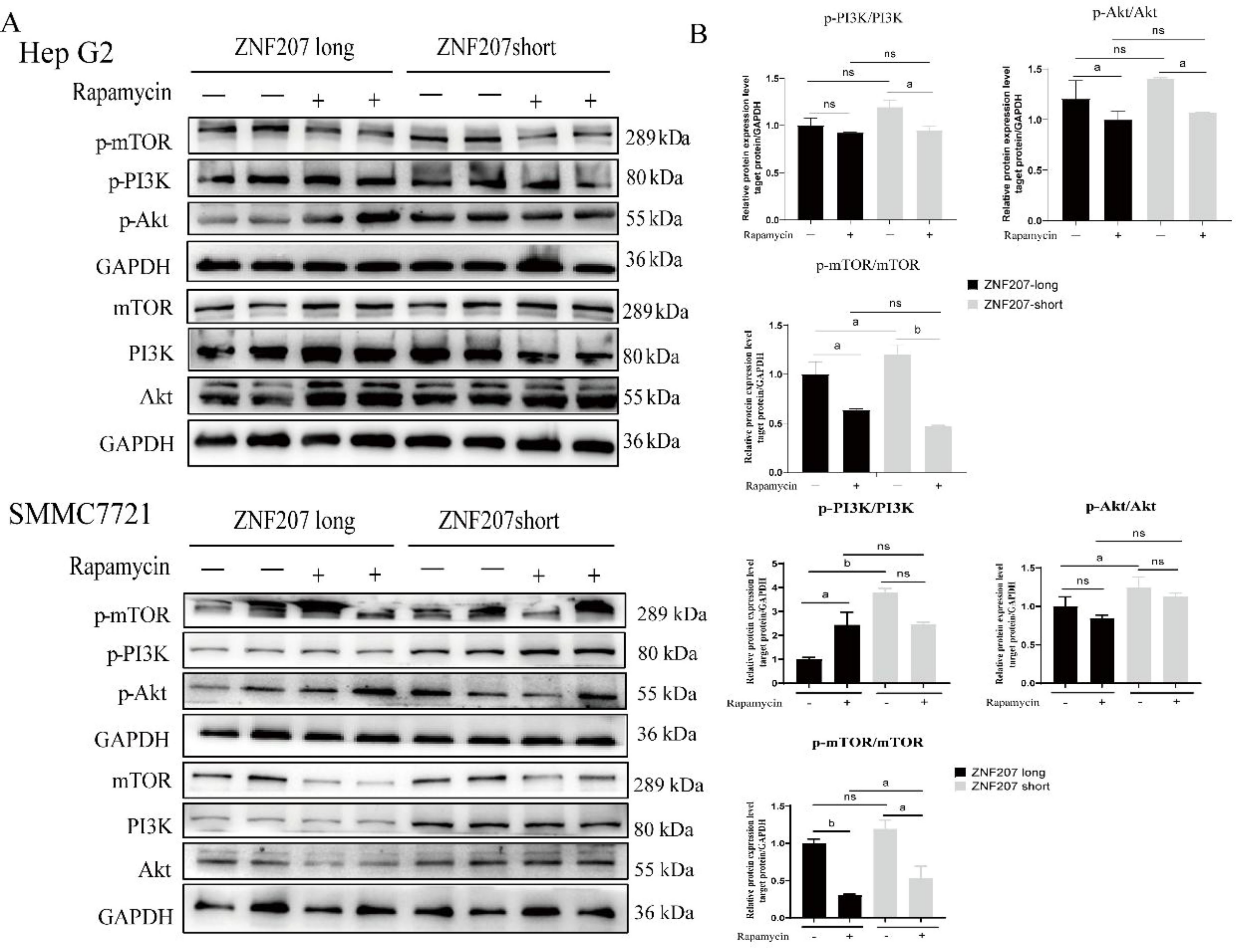
mTOR-mediated aberrant alternative splicing affects the PI3K/Akt signaling pathway in Hep G2. **(A)** Western blot analysis was used to assess the phosphorylation levels of PI3K, Akt and mTOR in HCC cells. **(B)** The densiometric analysis for PI3K, Akt and mTOR expression in HCC cells. ns, no significance, a: *p*<0.05, b: *p*<0.01. The original blots are presented in **Supplementary Figure S6D**.

As mTOR is a key downstream effector of the PI3K/Akt pathway and plays a crucial role in cell growth and proliferation, its inhibition, such as by rapamycin, is often targeted in cancer therapy due to the pathway’s frequent dysregulation in tumors. After transfecting Hep G2 cells with ZNF207-long and ZNF207-short for 36 h, the cells were treated with 8 μM rapamycin for 24 h. Western blot analysis showed that rapamycin effectively inhibited mTOR phosphorylation. Following the rapamycin treatment, there was a slight decrease in PI3K/Akt activity, but the difference in the PI3K/Akt signaling pathway expression between cells transfected with ZNF207-short and those with inhibited mTOR activity was not significant (**Figures 6A, B**).

## 4 Discussion

Aberrant alternative splicing (AS) profiles have been increasingly recognized for adding significant complexity to the oncogenic network and playing a pivotal role in the development and progression of cancers, including hepatocellular carcinoma (32–34). One potential explanation for the marked differences in splicing patterns between normal liver tissues and HCC is the varied expression of RNA-binding proteins and their splicing modalities. It has been observed that different liver disease models in mice display distinct profiles of trans-acting splicing factor 1 expression. While these variations in expression profiles do not directly alter the AS of genes, they are significantly associated with the survival outcomes in human HCC patients (35). This linkage suggests that changes in the expression levels of splicing factors (SFs) might be crucially linked to patient prognosis and could thus serve as a focal point for targeted therapeutic interventions (36). These findings underscore the importance of understanding the interplay between RNA-binding proteins and AS in modulating the oncogenic landscape of HCC, providing a promising avenue for the development of novel therapeutic strategies.

TCGA has revolutionized our comprehension of the genetic foundations of cancer through its utilization of high-throughput genomic analysis techniques (37). In this investigation, we observed a pronounced elevation of *hnRNPA1* expression in liver cancer tissues using the TCGA public database. Moreover, hnRNPA1 was found to be highly expressed in other cancer types, including cervical cancer, where it plays a crucial role in cancer cell growth, survival, and metabolic alterations, positioning it as an active driver of cancer progression (38). Previous research has indicated that hnRNPA1 can suppress the expression of the tumor suppressor gene *p53*, thus fostering the proliferation and metastasis of liver cancer cells (39). In contrast, long non-coding RNAs (lncRNAs) are known to modulate tumor metastasis and epithelial-mesenchymal transition (EMT) by interacting with hnRNPA1 and miRNAs (40). Latest research has showed that *hnRNPA1* positively regulates vaccinia-related kinase 1 (*VRK1*) translation via binding directly to the 3’ untranslated region (UTR) of *VRK1* mRNA, thus increasing cyclin D1 (*CCND1*) expression by *VRK1*-mediated phosphorylation of the cAMP response element-binding protein (CREB) (41). This study’s findings reveal that *hnRNPA1* not only directly modulates epithelial factors but also affects the alternative splicing (AS) of the transcription factor ZNF207. This interaction further influences tumor metastasis, providing fresh insights and a theoretical framework for understanding the role of *hnRNPA1* in the advancement of liver cancer. These observations highlight the complex interplay between *hnRNPA1*, its regulatory networks, and their impact on liver cancer pathogenesis, underscoring the potential of targeting *hnRNPA1* and its associated pathways in therapeutic strategies.

In this study, we have shown that *hnRNPA1* plays a pivotal role in enhancing cell proliferation and migration in HCC cells. Flow cytometry analysis revealed a marked increase in apoptosis rates following the downregulation of *hnRNPA1*. ZNF207, a member of the zinc finger protein family, is integral to various biological functions, including RNA splicing and protein translation. RIP analysis revealed *hnRNPA1* can bind to ZNF207 in Hep G2. Then software analysis enabled us to determine that the AS patterns of ZNF207 are altered in liver cancer tissues when *hnRNPA1* expression is reduced. Recent advancements in understanding the cancer-immune cycle have highlighted the anti-tumor immune responses, offering more precise therapeutic targets for HCC patients (42). Studies have explored immunosuppressive targets in HCC by integrating cancer-immune cycle scores with bioinformatics, identifying ZNF207 in this context. Nonetheless, the exact mechanisms through which ZNF207 contribute to the acceleration of HCC progression remain to be fully elucidated. Alternative splicing is a critical post-transcriptional regulation mechanism occurring in 95% of human exons, significantly increasing the diversity and complexity of the human proteome and complicating the disease processes (43). Different transcript isoforms exert distinct functions due to their varying exon-skipping patterns. In this study, we investigated the effects of transcript isoforms by constructing plasmids and transfecting them into HCC cells. Our findings demonstrated that ZNF207 produces two transcript isoforms of varying lengths by excluding exon 9. The shorter transcript isoforms were shown to promote proliferation and migration while inhibiting apoptosis in HCC cells. This observation is akin to findings in lung cancer, where inactivation of the splicing factor RBM10 alters the production of shorter transcript isoforms of the *EGFR* gene, impacting normal alveolar epithelial cell function and contributing to lung cancer progression (44). However, we were unable to detect the expression of these two isoforms in HCC tissues and adjacent normal tissues. Despite this limitation, our study demonstrated that reduced *hnRNPA1* expression favors the production of the shorter ZNF207 transcript isoforms, thereby enhancing cell proliferation and migration while suppressing apoptosis in HCC cells. In future studies, we aim to further investigate the expression differences and underlying mechanisms of these two transcript isoforms in normal liver tissues and liver cancer, to better understand their roles in HCC progression.

Previous results confirmed that *hnRNPA1* affects the growth and survival of cancer cells and becomes active proteins in cancer cells. The PI3K/Akt/mTOR pathway is an intracellular pathway directly associated with cell proliferation, growth, and cancer (45). In this study, we found that *hnRNPA1* promotes the expression of mTOR, which activates PI3K/Akt and affects cell proliferation and migration. Concurrently, the shorter transcript isoforms of ZNF207 accelerate PI3K/Akt/mTOR pathway activation and increase hnRNPA1 expression. Conversely, the longer transcripts of ZNF207 results in the opposite effect. Overall, the interaction between alternative splicing and the PI3K/Akt/mTOR signaling pathways is bidirectional, with each pathway influencing and regulating the other. These processes play crucial roles in the regulation of fundamental biological functions including cell growth, metabolism, and survival. This interplay introduces a novel layer of intricacy to intracellular signaling networks and offers fresh perspectives for investigating the pathogenesis and potential therapeutic targets of associated diseases.

In conclusion, our study elucidated the pivotal role of *hnRNPA1* in regulating the progression of HCC through its influence on the alternative splicing of ZNF207. Specifically, we demonstrated that *hnRNPA1* modulates the splicing of exon 9 in ZNF207, which in turn affects key signaling pathways, notably the PI3K/Akt/mTOR pathway, that governs cell proliferation and survival. The silencing of *hnRNPA1* resulted in reduced proliferation and increased apoptosis in Hep G2 cells, confirming its critical function in cancer cell dynamics. These findings not only enhance our understanding of the molecular mechanisms underlying HCC but also highlight potential therapeutic targets for disrupting the hnRNPA1-ZNF207 interaction. By targeting this pathway, future therapies might better manage or even inhibit the aggressive nature of HCC, offering hope for improved clinical outcomes.

## 5 Limitations of the study

We provide extensive evidence that *hnRNPA1* regulates the progression of HCC by influencing the alternative splicing of ZNF207. However, it should be noted that our assessment of ZNF207’s alternative splicing may not be entirely accurate or fully standardized. In this work, we have specifically analyzed gene expression profiles from 424 tissue samples extracted from TCGA database and concluded *hnRNPA1* was high expression in HCC. However, the samples used for immunohistochemistry were relatively small, which may limit to fully demonstrate the expression of hnRNPA1 in normal or tumor tissues. Although we examined the protein expression of hnRNPA1 in different cells, we have also not tested the protein expression of hnRNPA1 in tumor tissue. Further studies will provide larger samples to enhance the reliability of the findings.

## Data Availability

The datasets presented in this study can be found in online repositories. The names of the repository/repositories and accession number(s) can be found in the article/**Supplementary Material**.

## Supplementary material

Supplementary Figure 1 The expression levels of *hnRNPA1* in HCC. The original blots of **Figure 1D** illustrate the following: the left membrane was stained with anti-β-actin antibody, the right membrane was probed with anti-hnRNPA1 antibody.

Supplementary Figure 2 Knockdown of *hnRNPA1* influences Hep G2 cell growth. **(A)** The expression of *hnRNPA1* in Hep G2. b: *p* < 0.01**(B)** MTT assay showed that *hnRNPA1* knockdown inhibited Hep G2 proliferation. a: *p* < 0.05. **(C)** Cell clone formation assay showed that *hnRNPA1* knockdown inhibited Hep G2 proliferation. a: *p* < 0.05. **(D)** Wound healing assay showed that *hnRNPA1* knockdown inhibited Hep G2 migration. b: *p* < 0.01. Scale bar, 200 μm. **(E)** Western blot analysis showed that *hnRNPA1* knockdown slowed down the process of apoptosis. ns, no significance, a: *p* < 0.05, b: *p* < 0.01. **(F)** The original blots of **(E)** illustrate the following: the top membrane was stained with anti-GAPDH (top) and anti-β-tubulin (down) antibody, the second membranes were probed with anti-Bcl2 (left) and anti-Bax antibody (right), and the third membranes were probed with anti-Caspase3 (left) and anti-cleaved Caspase3 antibody (right), the low membranes were probed with anti-hnRNPA1 (left) and anti-β-tubulin antibody (right).

Supplementary Figure 3 Knockdown of *hnRNPA1* inhibits HCC cells proliferation and migration. **(A)** The original blots of **Figure 3A** illustrate the following: the top membrane was stained with anti-β-tubulin antibody (left) and anti-hnRNPA1 antibody (right) in Hep G2, the down membrane represents the top membrane was stained with anti-β-tubulin antibody (left) and anti-hnRNPA1 antibody (right) in SMMC7721. **(B)** Relative mRNA expression of genes involved in cell proliferation, cell apoptosis and vascular endothelial growth. *ns*, no significance, a: *p* < 0.05, b: *p* < 0.01, c: *p* < 0.005, d: *p* < 0.001.

Supplementary Figure 4 ZNF207-short but not ZNF207-long promoted cell proliferation and migration. **(A)** The full gel electrophoresis shows ZNF207 transcription level in knockdown of *hnRNPA1* in Hep G2 cell lines. A representative assay illustrated the change of alternative splicing, as determined by the TBE analysis. The top gel was the *β-actin* PCR products (~139 bp) of control and knockdown *hnRNPA1* in Hep G2. The down gel was the ZNF207 transcripts PCR products (~236bp) were digested into one fragment (~144bp), indicative of skipped exons. **(B)** Overlapping PCR flowchart. **(C)** Mutant plasmids for ZNF207-short and ZNF207-long.

Supplementary Figure 5 ZNF207-short but not ZNF207-long promotes proliferation and migration. **(A)** Flow cytometry experiments showed that ZNF207-short, inhibited apoptosis in HCC cells. **(B)** The original blots of **Figure 5E** illustrate the following: the top membrane was stained with anti-Bax antibody (left) and anti-Bcl2 antibody (right) in Hep G2, the middle membrane was probed with anti-Caspase3 antibody (left) and anti-Cleaved Caspase3 antibody (right) in Hep G2. The lower membrane was probed with anti-GAPDH antibody in Hep G2. The rest membranes in SMMC 7721 were probed with antibody in equal order.

Supplementary Figure 6 ZNF207 affects the PI3K/Akt signaling pathway in Hep G2. **(A)** Western blot analysis of PI3K, Akt and mTOR in Hep G2. **(B)** The densiometric analysis for PI3K, Akt and mTOR in Hep G2. *ns*, no significance, a: *p* < 0.05, b: *p* < 0.01. **(C)** The original blots of **(A)** illustrate the following: the top membrane was stained with anti-mTOR & p-mTOR antibody (left) and anti-GAPDH antibody (right) in Hep G2, the middle membrane was probed with anti-PI3K antibody (left) and p-PI3K antibody (right) in Hep G2. The lower membrane was probed with anti-Akt antibody (left) and p-Akt antibody (right) in Hep G2. **(D)** The original blots of **Figure 6A** illustrate the following: the top membrane was stained with anti-mTOR & p-mTOR antibody (left) and anti-GAPDH antibody (right) in Hep G2, the middle membrane was probed with anti-PI3K antibody (left) and p-PI3K antibody (right) in Hep G2. The lower membrane was probed with anti-Akt antibody (left) and p-Akt antibody (right) in Hep G2. The rest membranes in SMMC 7721 were probed with antibody in equal order.

## References

[1] Llovet JM Pinyol R Yarchoan M Singal AG Marron TU Schwartz M. Adjuvant and neoadjuvant immunotherapies in hepatocellular carcinoma. Nat Rev Clin Oncol. (2024) 21:294–311. doi: 10.1038/s41571-024-00868-0 38424197 PMC11984461

[2] Fuster-Anglada C Mauro E Ferrer-Fàbrega J Caballol B Sanduzzi-Zamparelli M Bruix J. Histological predictors of aggressive recurrence of hepatocellular carcinoma after liver resection. J Hepatol. (2024) 81:995–1004. doi: 10.1016/j.jhep.2024.06.018 38925272

[3] Li S Qu Y Liu L Wang C Yuan L Bai H. Tumour-derived exosomes in liver metastasis: A Pandora's box. Cell Prolif. (2023) 56:e13452. doi: 10.1111/cpr.v56.10 36941028 PMC10542622

[4] Xue R Zhang Q Cao Q Kong R Xiang X Liu H. Liver tumour immune microenvironment subtypes and neutrophil heterogeneity. Nature. (2022) 612:141–7. doi: 10.1038/s41586-022-05400-x 36352227

[5] Mo A Lin B Chen D. Efficacy of sequential TACE on primary hepatocellular carcinoma with microvascular invasion after radical resection: a systematic review and meta-analysis. World J Surg Oncol. (2023) 21:277. doi: 10.1186/s12957-023-03160-0 37667375 PMC10478229

[6] Sun Y Wu P Zhang Z Wang Z Zhou K Song M. Integrated multi-omics profiling to dissect the spatiotemporal evolution of metastatic hepatocellular carcinoma. Cancer Cell. (2024) 42:135–156.e17. doi: 10.1016/j.ccell.2023.11.010 38101410

[7] Wani AK Akhtar N Sharma A El-Zahaby SA. Fighting carcinogenesis with plant metabolites by weakening proliferative signaling and disabling replicative immortality networks of rapidly dividing and invading cancerous cells. Curr Drug Delivery. (2023) 20:371–86. doi: 10.2174/1567201819666220414085606 35422214

[8] Wani AK Akhtar N Mir TUG Singh R Jha PK Mallik SK. Targeting apoptotic pathway of cancer cells with phytochemicals and plant-based nanomaterials. Biomolecules. (2023) 13. doi: 10.3390/biom13020194 PMC9953589 36830564

[9] Corley M Burns MC Yeo GW. How RNA-binding proteins interact with RNA: molecules and mechanisms. Mol Cell. (2020) 78:9–29. doi: 10.1016/j.molcel.2020.03.011 32243832 PMC7202378

[10] Hentze MW Castello A Schwarzl T Preiss T. A brave new world of RNA-binding proteins. Nat Rev Mol Cell Biol. (2018) 19:327–41. doi: 10.1038/nrm.2017.130 29339797

[11] Ule J Blencowe BJ. Alternative splicing regulatory networks: functions, mechanisms, and evolution. Mol Cell. (2019) 76:329–45. doi: 10.1016/j.molcel.2019.09.017 31626751

[12] Lee SE Alcedo KP Kim HJ Snider NT. Alternative splicing in hepatocellular carcinoma. Cell Mol Gastroenterol Hepatol. (2020) 10:699–712. doi: 10.1016/j.jcmgh.2020.04.018 32389640 PMC7490524

[13] Green MR. Pre-mRNA splicing. Annu Rev Genet. (1986) 20:671–708. doi: 10.1146/annurev.ge.20.120186.003323 2880558

[14] Chaudhury A Chander P Howe PH. Heterogeneous nuclear ribonucleoproteins (hnRNPs) in cellular processes: Focus on hnRNP E1's multifunctional regulatory roles. RNA (New York N.Y.). (2010) 16:1449–62. doi: 10.1261/rna.2254110 PMC2905745 20584894

[15] Martinez-Contreras R Cloutier P Shkreta L Fisette JF Revil T Chabot B. hnRNP proteins and splicing control. Adv Exp Med Biol. (2007) 623:123–47. doi: 10.1007/978-0-387-77374-2_8 18380344

[16] Kędzierska H Piekiełko-Witkowska A. Splicing factors of SR and hnRNP families as regulators of apoptosis in cancer. Cancer Lett. (2017) 396:53–65. doi: 10.1016/j.canlet.2017.03.013 28315432

[17] Paronetto MP Achsel T Massiello A Chalfant CE Sette C. The RNA-binding protein Sam68 modulates the alternative splicing of Bcl-x. J Cell Biol. (2007) 176:929–39. doi: 10.1083/jcb.200701005 PMC2064079 17371836

[18] Clarke JP Thibault PA Fatima S Salapa HE Kalyaanamoorthy S Ganesan A. Sequence- and structure-specific RNA oligonucleotide binding attenuates heterogeneous nuclear ribonucleoprotein A1 dysfunction. Front Mol Biosci. (2023) 10:1178439. doi: 10.3389/fmolb.2023.1178439 37426420 PMC10325567

[19] Drummond E Pires G Macmurray C Askenazi M Nayak S Bourdon M. Phosphorylated tau interactome in the human Alzheimer's disease brain. Brain. (2020) 143:2803–17. doi: 10.1093/brain/awaa223 PMC7526722 32812023

[20] Jiang H He X Wang S Jia J Wan Y Wang Y. A microtubule-associated zinc finger protein, BuGZ, regulates mitotic chromosome alignment by ensuring Bub3 stability and kinetochore targeting. Dev Cell. (2014) 28:268–81. doi: 10.1016/j.devcel.2013.12.013 PMC3927447 24462186

[21] Wang X Zhou T Chen X Wang Y Ding Y Tu H. System analysis based on the cancer-immunity cycle identifies ZNF207 as a novel immunotherapy target for hepatocellular carcinoma. J Immunother Cancer. (2022) 10. doi: 10.1136/jitc-2021-004414 PMC8900045 35246476

[22] Zhou C Li N. Expression of ZNF207 in hepatocellular carcinoma and its significance. Zhong Nan Da Xue Xue Bao Yi Xue Ban. (2019) 44:406–12. doi: 10.11817/j.issn.1672-7347.2019.04.010 31113916

[23] Llovet JM Montal R Sia D Finn RS. Molecular therapies and precision medicine for hepatocellular carcinoma. Nat Rev Clin Oncol. (2018) 15:599–616. doi: 10.1038/s41571-018-0073-4 30061739 PMC12452113

[24] Shang L Jiang W Zhang J Wu W. P4HA2 promotes occurrence and progression of liver cancer by regulating the PI3K/Akt/mTOR signaling pathway. Nan Fang Yi Ke Da Xue Xue Bao = J South Med Univ. (2022) 42:665–72. doi: 10.12122/j.issn.1673-4254.2022.05.06 PMC9178641 35673909

[25] Panwar V Singh A Bhatt M Tonk RK Azizov S Raza AS. Multifaceted role of mTOR (mammalian target of rapamycin) signaling pathway in human health and disease. Signal Transduct Target Ther. (2023) 8:375. doi: 10.1038/s41392-023-01608-z 37779156 PMC10543444

[26] Artoni F Grützmacher N Demetriades C. Unbiased evaluation of rapamycin's specificity as an mTOR inhibitor. Aging Cell. (2023) 22:e13888. doi: 10.1111/acel.13888 37222020 PMC10410055

[27] Voskoboinik I Thia M-C Trapani JA. A functional analysis of the putative polymorphisms A91V and N252S and 22 missense perforin mutations associated with familial hemophagocytic lymphohistiocytosis. Blood. (2005) 105:4700–6. doi: 10.1182/blood-2004-12-4935 15755897

[28] Muñoz-Couselo E Adelantado EZ Ortiz C García JS Perez-Garcia J. NRAS-mutant melanoma: current challenges and future prospect. OncoTargets Ther. (2017) 10:3941–7. doi: 10.12122/j.issn.1673-4254.2022.05.06 PMC5558581 28860801

[29] Lu M Tan L Zhou X-G Yang Z-L Zhu Q Chen J-N. Secoisolariciresinol Diglucoside Delays the Progression of Aging-Related Diseases and Extends the Lifespan of Caenorhabditis elegans via DAF-16 and HSF-1. Oxid Med Cell Longevity. (2020) 2020:1293935. doi: 10.1155/2020/1293935 PMC7378611 32733632

[30] Moshiri A Puppo M Rossi M Gherzi R Briata P. Resveratrol limits epithelial to mesenchymal transition through modulation of KHSRP/hnRNPA1-dependent alternative splicing in mammary gland cells. Biochim Biophys Acta Gene Regul Mech. (2017) 1860:291–8. doi: 10.1016/j.bbagrm.2017.01.001 28088441

[31] Qi T Qu Q Li G Wang J Zhu H Yang Z. Function and regulation of the PEA3 subfamily of ETS transcription factors in cancer. Am J Cancer Res. (2020) 10:3083–105. PMC7642666 33163259

[32] Kahles A Lehmann KV Toussaint NC Hüser M Stark SG Sachsenberg T. Comprehensive analysis of alternative splicing across tumors from 8,705 patients. Cancer Cell. (2018) 34:211–224.e6. doi: 10.1016/j.ccell.2018.07.001 30078747 PMC9844097

[33] Climente-González H Porta-Pardo E Godzik A Eyras E. The functional impact of alternative splicing in cancer. Cell Rep. (2017) 20:2215–26. doi: 10.1016/j.celrep.2017.08.012 28854369

[34] Montes M Sanford BL Comiskey DF Chandler DS. RNA splicing and disease: animal models to therapies. Trends Genet. (2019) 35:68–87. doi: 10.1016/j.tig.2018.10.002 30466729 PMC6339821

[35] Xu K Wu T Xia P Chen X Yuan Y. Alternative splicing: a bridge connecting NAFLD and HCC. Trends Mol Med. (2023) 29:859–72. doi: 10.1016/j.molmed.2023.07.001 37487782

[36] Jobbins AM Yu S Paterson HAB Maude H Kefala-Stavridi A Speck C. Pre-RNA splicing in metabolic homeostasis and liver disease. Trends Endocrinol Metab. (2023) 34:823–37. doi: 10.1016/j.tem.2023.08.007 37673766

[37] Cheishvili D Wong C Karim MM Kibria MG Jahan N Das PC. A high-throughput test enables specific detection of hepatocellular carcinoma. Nat Commun. (2023) 14:3306. doi: 10.1038/s41467-023-39055-7 37286539 PMC10247794

[38] Francies FZ Bassa S Chatziioannou A Kaufmann AM Dlamini Z. Splicing genomics events in cervical cancer: insights for phenotypic stratification and biomarker potency. Genes (Basel). (2021) 12. doi: 10.3390/genes12020130 PMC7909518 33498485

[39] Wojtyś W Oroń M. How driver oncogenes shape and are shaped by alternative splicing mechanisms in tumors. Cancers (Basel). (2023) 15. doi: 10.3390/cancers15112918 PMC10251868 37296881

[40] Wen Z Lian L Ding H Hu Y Xiao Z Xiong K. LncRNA ANCR promotes hepatocellular carcinoma metastasis through upregulating HNRNPA1 expression. RNA Biol. (2020) 17:381–94. doi: 10.1080/15476286.2019.1708547 PMC6999620 31868085

[41] Ryu HG Jung Y Lee N Seo JY Kim SW Lee KH. HNRNP A1 promotes lung cancer cell proliferation by modulating VRK1 translation. Int J Mol Sci. (2021) 22. doi: 10.3390/ijms22115506 PMC8197126 34071140

[42] Chen DS Mellman I. Oncology meets immunology: the cancer-immunity cycle. Immunity. (2013) 39:1–10. doi: 10.1016/j.immuni.2013.07.012 23890059

[43] Mehterov N Kazakova M Sbirkov Y Vladimirov B Belev N Yaneva G. Alternative RNA splicing-the trojan horse of cancer cells in chemotherapy. Genes (Basel). (2021) 12. doi: 10.3390/genes12071085 PMC8306420 34356101

[44] Nanjo S Wu W Karachaliou N Blakely CM Suzuki J Chou YT. Deficiency of the splicing factor RBM10 limits EGFR inhibitor response in EGFR-mutant lung cancer. J Clin Invest. (2022) 132. doi: 10.1172/JCI145099 PMC9246391 35579943

[45] Man J Zhou W Zuo S Zhao X Wang Q Ma H. TANGO1 interacts with NRTN to promote hepatocellular carcinoma progression by regulating the PI3K/AKT/mTOR signaling pathway. Biochem Pharmacol. (2023) 213:115615. doi: 10.1016/j.bcp.2023.115615 37211171

