# Supplementary material for: Downregulation of hnRNPA1 inhibits hepatocellular carcinoma cell progression by modulating alternative splicing of ZNF207 exon 9
Source: Front Oncol. 2025 Jan 6;14:1517459. doi: 10.3389/fonc.2024.1517459 (PMC11743940; doi:10.3389/fonc.2024.1517459)
Supplement: Supplementary Figure 1 — The expression levels of hnRNPA1 in HCC. The original blots of Figure 1D illustrate the following: the left membrane was stained with anti-β-actin antibody, the right membrane was probed with anti-hnRNPA1 antibody. [file Image1.pdf]

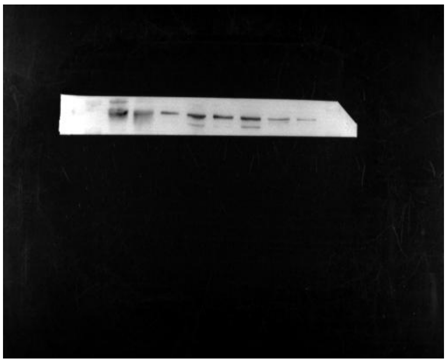

Probed with anti-β-actin antibody

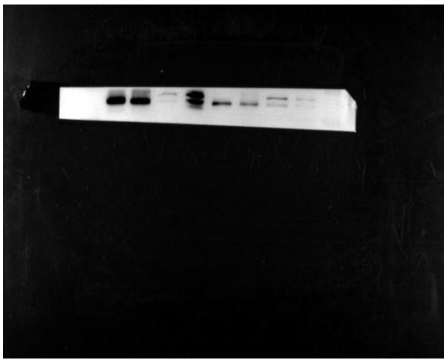

Probed with anti-hnRNPA1 antibody

Supplementary Figure 1. The expression levels of *hnRNPA1* in HCC. The original blots of Figure 1D illustrate the following: the left membrane was stained with anti-β-actin antibody, the right membrane was probed with anti-hnRNPA1 antibody.

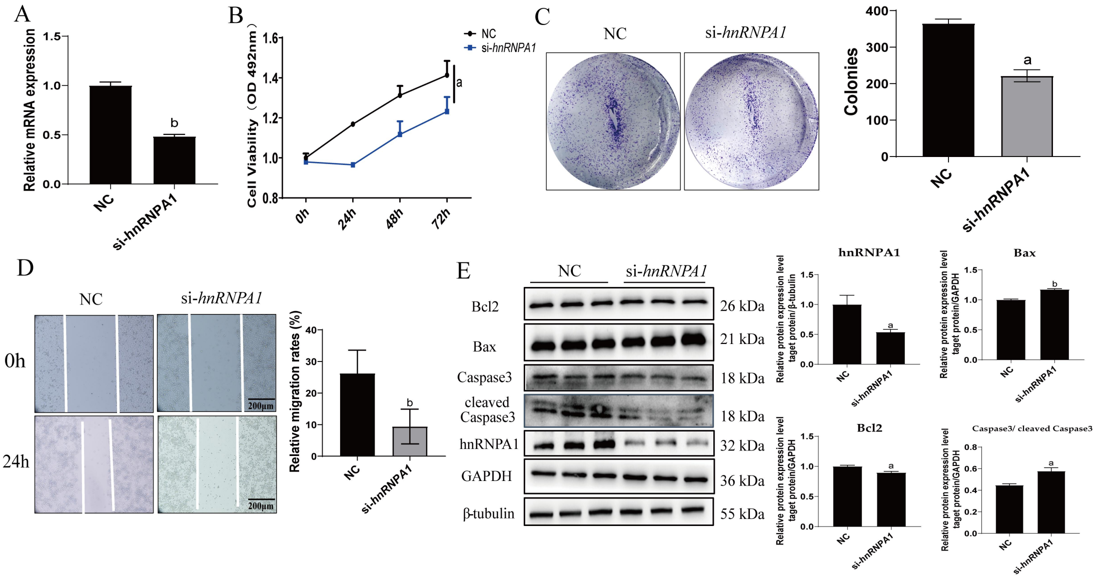

F

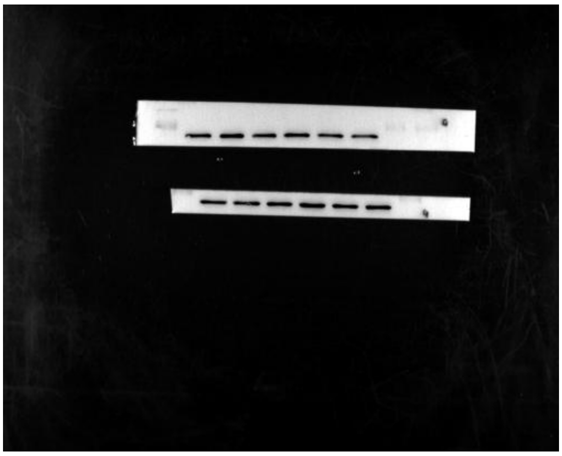

Probed with anti-GAPDH antibody (up)

Probed with anti-β-tubulin antibody

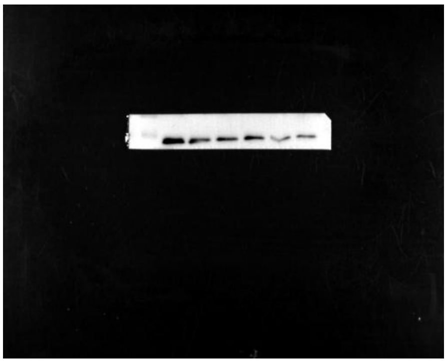

Probed with anti-Bcl2 antibody

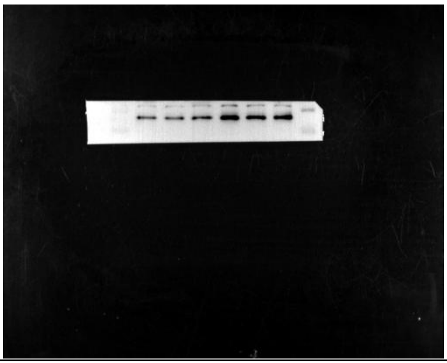

Probed with anti-Bax antibody

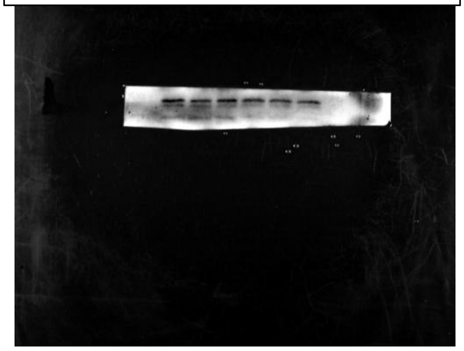

Probed with anti-Caspase3 antibody

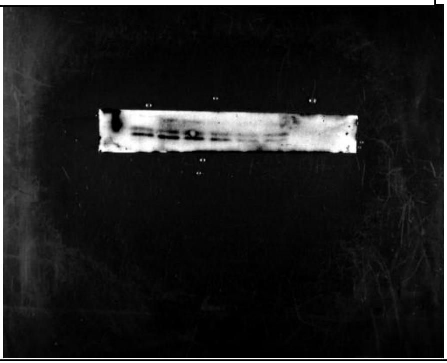

Probed with anti-Cleaved Caspase3

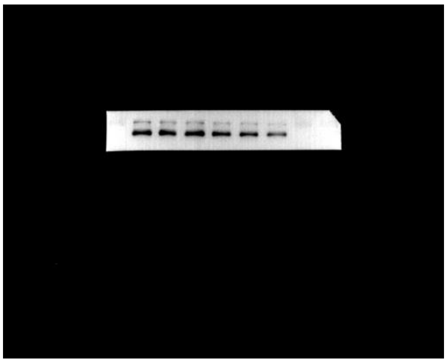

Probed with anti-hnRNPA1 antibody

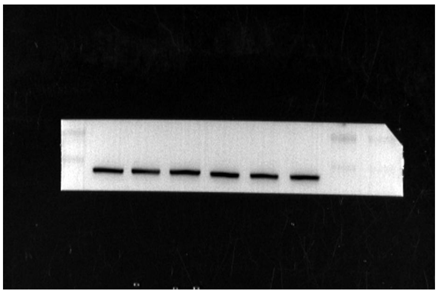

Probed with anti-β-tubulin antibody

Supplementary Figure 2. Knockdown of *hnRNPA1* influences Hep G2 cell growth. (A) The expression of *hnRNPA1* in Hep G2. b: $p < 0.01$(B) MTT assay showed that *hnRNPA1* knockdown inhibited Hep G2 proliferation. a: $p < 0.05$. (C) Cell clone formation assay showed that *hnRNPA1* knockdown inhibited Hep G2 proliferation. a: $p < 0.05$. (D) Wound healing assay showed that *hnRNPA1* knockdown inhibited Hep G2 migration. b: $p < 0.01$. Scale bar, 200 μm. (E) Western blot analysis showed that *hnRNPA1* knockdown slowed down the process of apoptosis. ns, no significance, a: $p < 0.05$, b:$p < 0.01$. (F) The original blots of Supplementary Figure S2E illustrate the following: the top membranes were stained with anti-GAPDH (top) and anti-β-tubulin (down) antibody, the second membranes were probed with anti-Bcl2 (left) and anti-Bax antibody (right), and the third membranes were probed with anti-Caspase3 (left) and anti-cleaved Caspase3 antibody (right), the low membranes were probed

with anti-hnRNPA1 (left) and anti-β-tubulin antibody (right) .

A

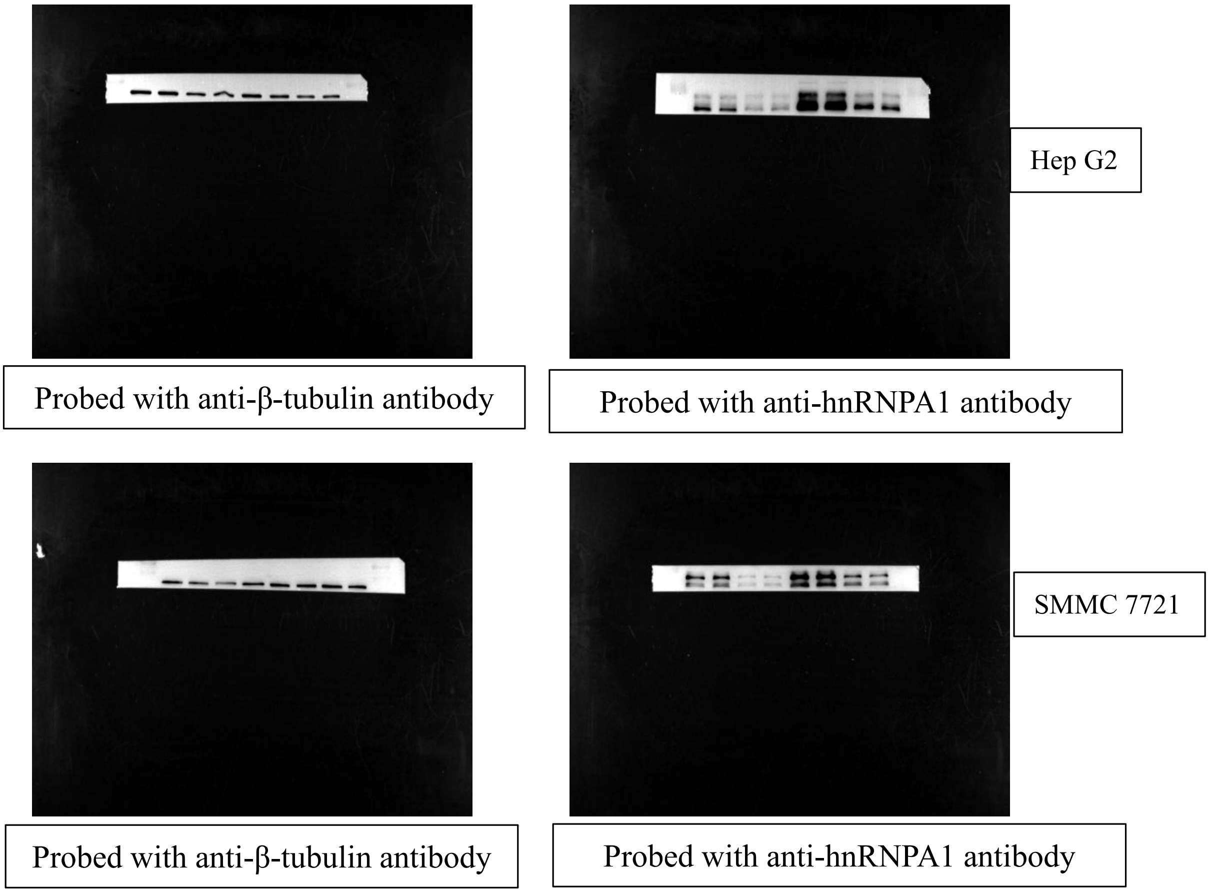

B

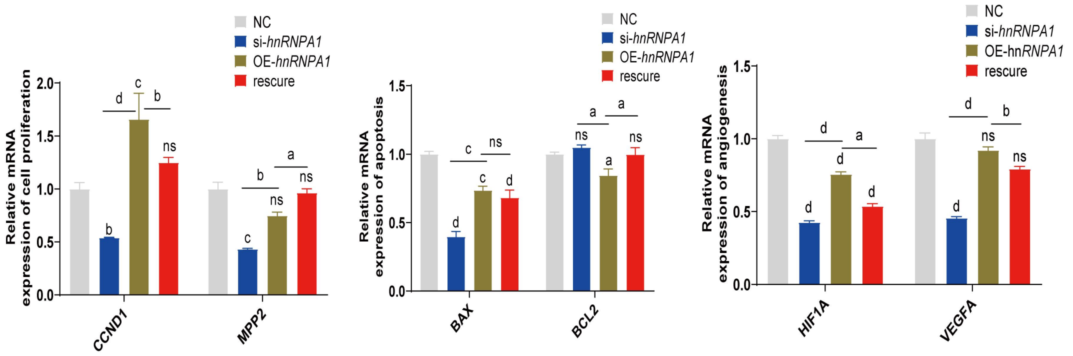

Supplementary Figure 3. Knockdown of *hnRNPA1* inhibits HCC cells proliferation and migration. (A) The original blots of Figure 3A illustrate the following: the top membrane was stained with anti-β-tubulin antibody (left) and anti-hnRNPA1 antibody (right) in Hep G2, the down membrane represents he top membrane was stained with anti-β-tubulin antibody (left) and anti-hnRNPA1 antibody (right) in SMMC7721. (B) Relative mRNA expression of genes involved in cell proliferation, cell apoptosis and vascular endothelial growth. *ns*, no significance, a: $p < 0.05$, b: $p < 0.01$, c: $p < 0.005$,d: $p < 0.001$.

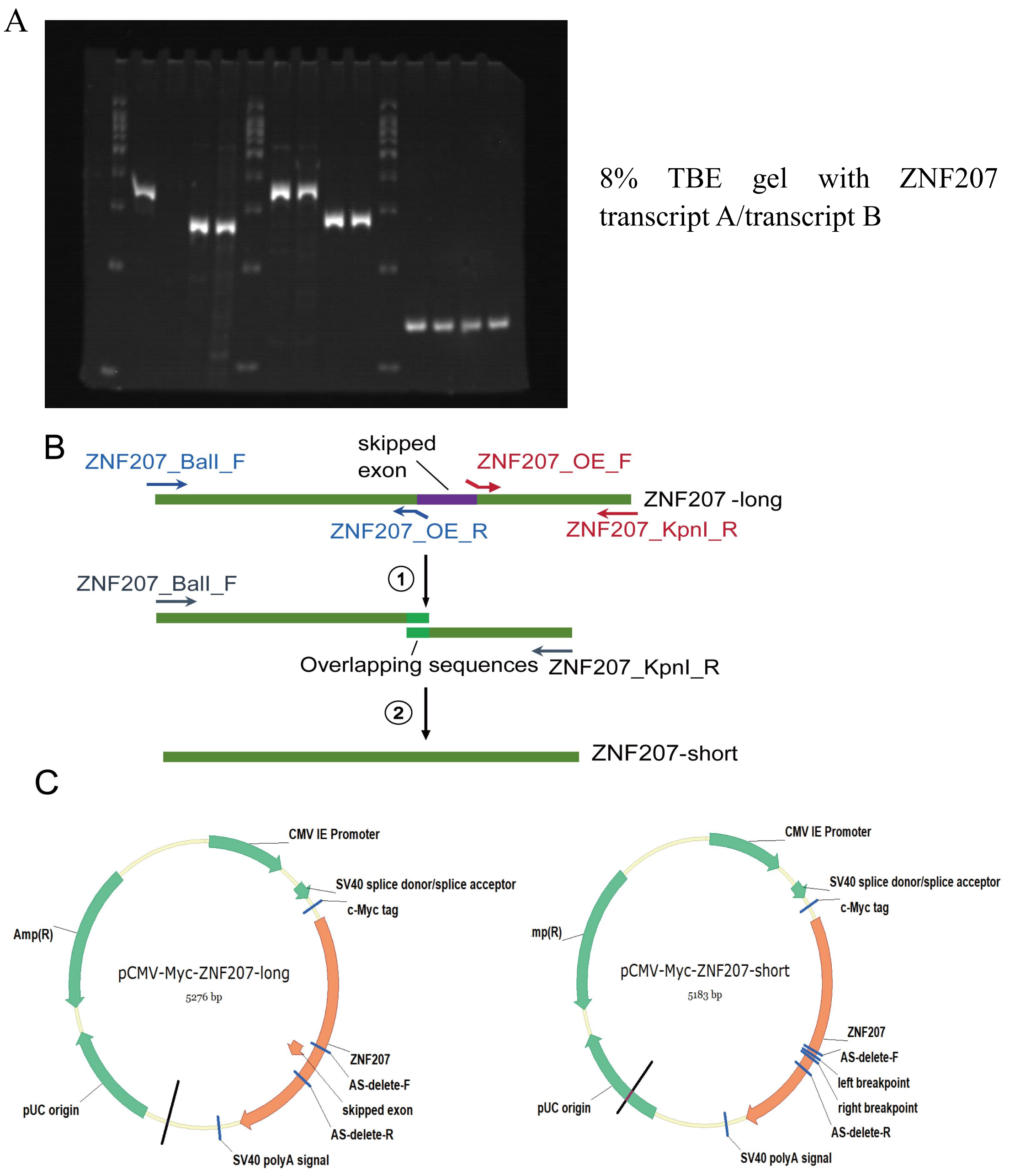

Supplementary Figure 4. ZNF207-short but not ZNF207-long promoted cell proliferation and migration. (A) The full gel electrophoresis shows ZNF207 transcription level in knockdown of *hnRNPA1* in Hep G2 cell lines. A representative assay illustrated the change of the alternative splicing, as determined by the TBE analysis. The top gel was the *β-actin* PCR products(~139 bp) of control and knockdown *hnRNPA1* in Hep G2. The down gel was the ZNF207 transcripts PCR products (~236bp) were digested into one fragment (~144bp), indicative of skipped exons. (B) Overlapping PCR flowchart. (C) Mutant plasmids for ZNF207-short and ZNF207-long.

A

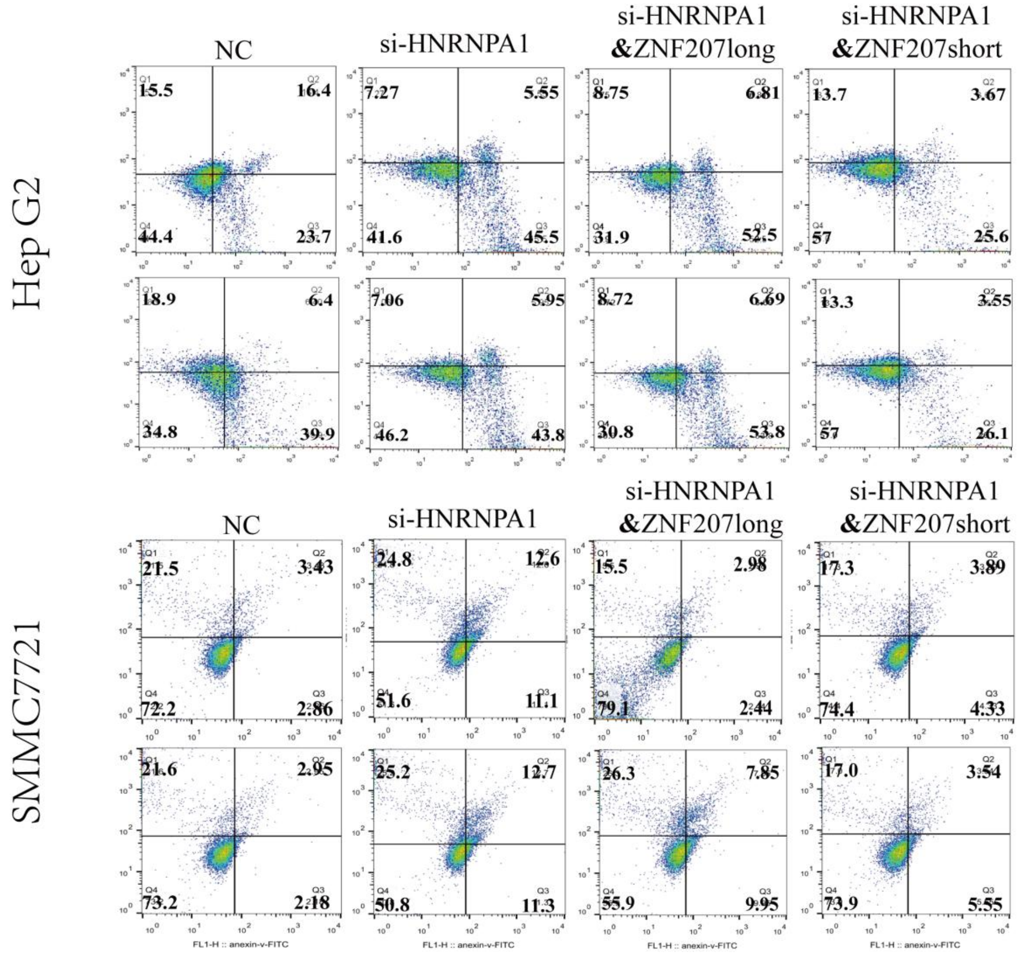

B

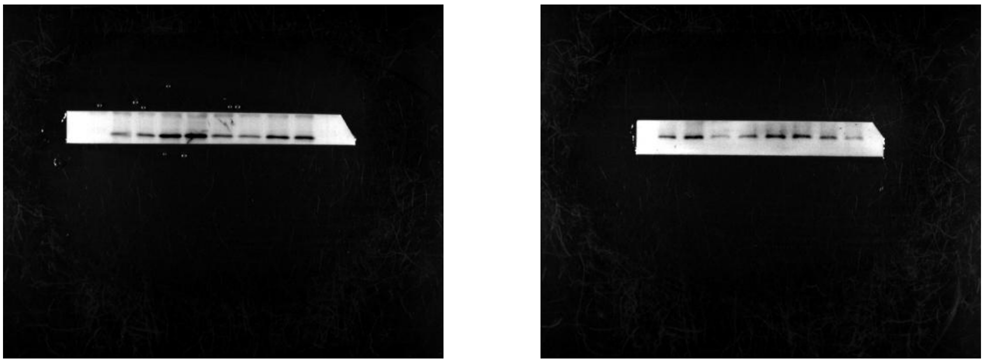

Hep G2

Probed with anti-Bax antibody

Probed with anti-Bcl2 antibody

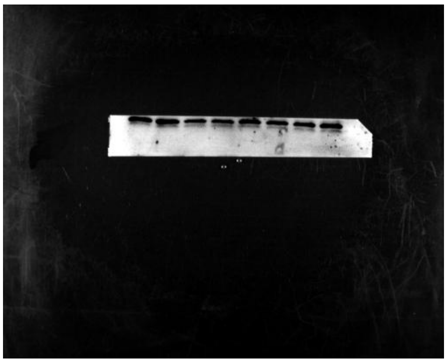

Probed with anti-Caspase3 antibody

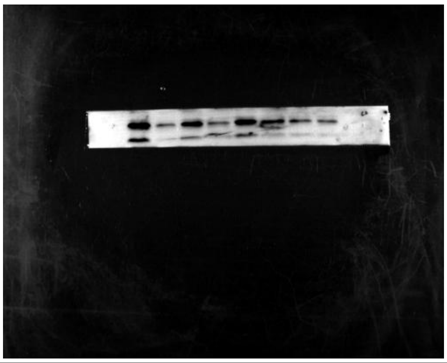

Hep G2

Probed with anti-Cleaved Caspase3 antibody

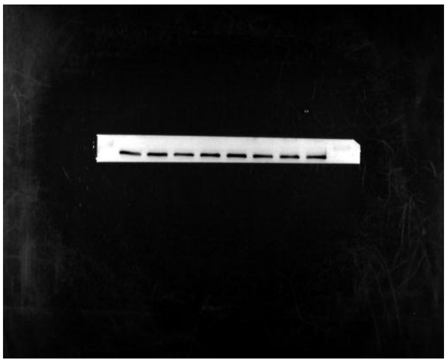

Hep G2

Probed with anti-GAPDH antibody

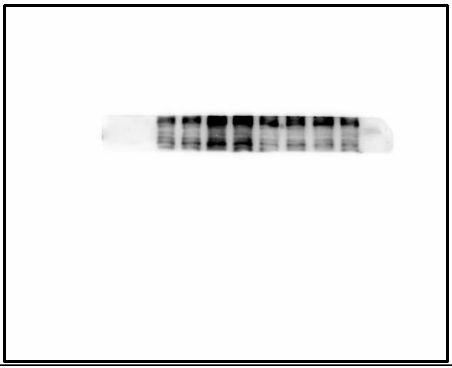

Probed with anti-Bax antibody

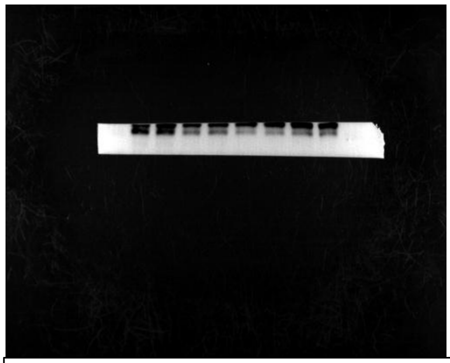

SMMC 7721

Probed with anti-Bcl2 antibody

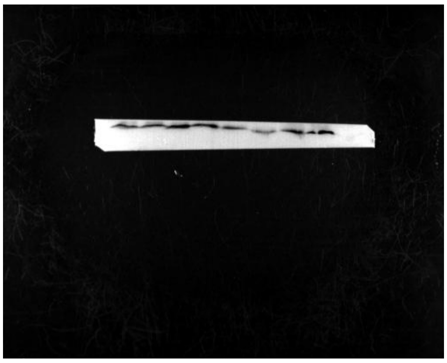

Probed with anti-Caspase3 antibody

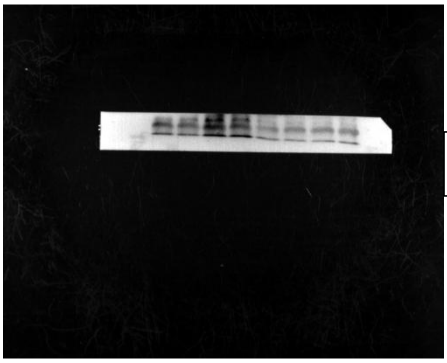

SMMC 7721

Probed with anti-Cleaved Caspase3 antibody

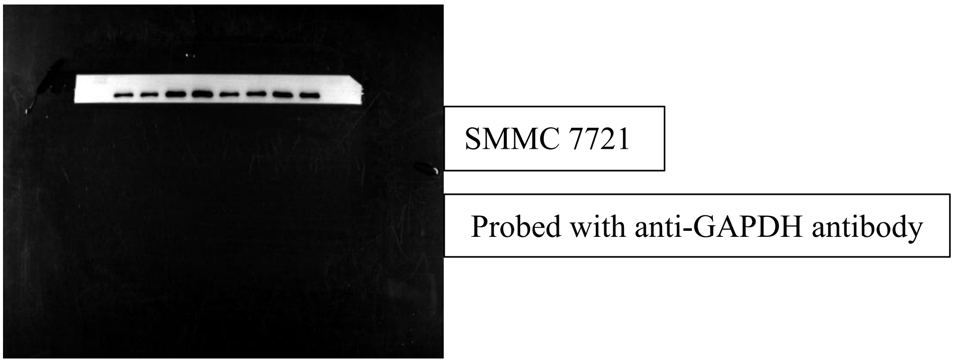

Supplementary Figure 5. ZNF207-short but not ZNF207-long promotes proliferation and migration. (A) Flow cytometry experiments showed that ZNF207-short inhibited apoptosis in HCC cells. (B) The original blots of Figure 5E illustrate the following: the top membrane was stained with anti-Bax antibody (left) and anti-Bcl2 antibody (right) in Hep G2, the middle membrane was probed with anti-Caspase3 antibody (left) and anti-Cleaved Caspase3 antibody (right) in Hep G2. The lower membrane was probed with anti-GAPDH antibody in Hep G2. The rest membranes in SMMC 7721 were probed with antibody in equal order.

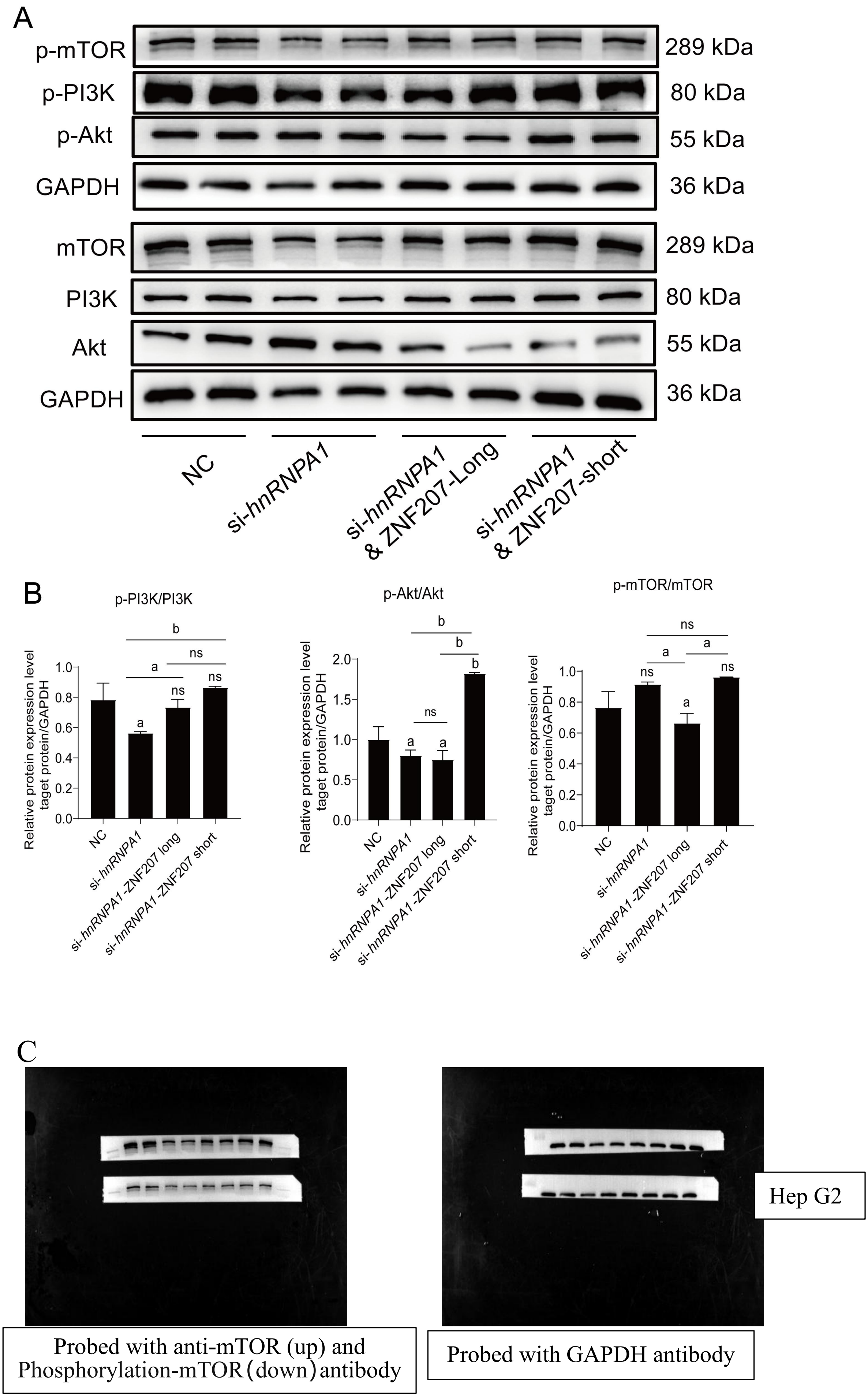
A
p-mTOR
289 kDa
p-PI3K
80 kDa
p-Akt
55 kDa
GAPDH
36 kDa
mTOR
289 kDa
PI3K
80 kDa
Akt
55 kDa
GAPDH
36 kDa
NC
si-hnRNPA1
si-hnRNPA1 & ZNF207-Long
si-hnRNPA1 & ZNF207-short
B
p-PI3K/PI3K
p-Akt/Akt
p-mTOR/mTOR
Relative protein expression level taget protein/GAPDH
NC
si-hnRNPA1
si-hnRNPA1-ZNF207 long
si-hnRNPA1-ZNF207 short
C
Hep G2
Probed with anti-mTOR (up) and Phosphorylation-mTOR (down) antibody
Probed with GAPDH antibody

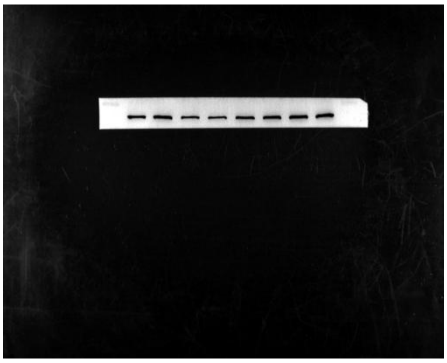

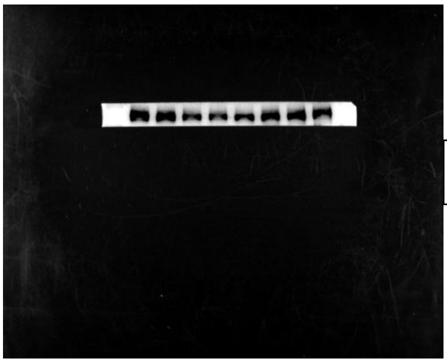

Hep G2

Probed with anti-PI3K antibody

Probed with anti-Phosphorylation-PI3K antibody

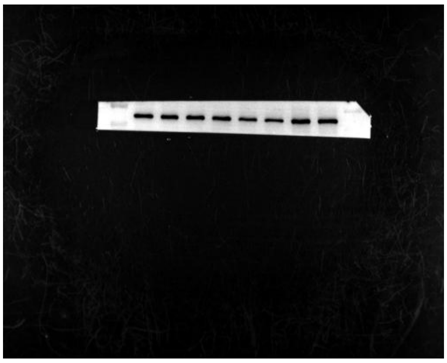

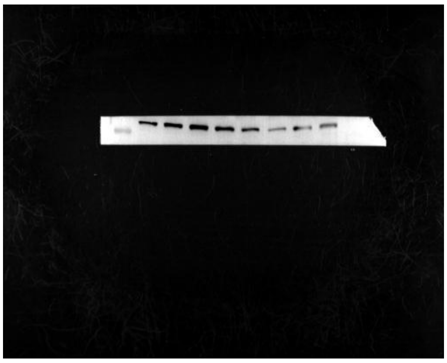

Hep G2

Probed with anti-Akt antibody

Probed with anti-Phosphorylation-Akt antibody

D

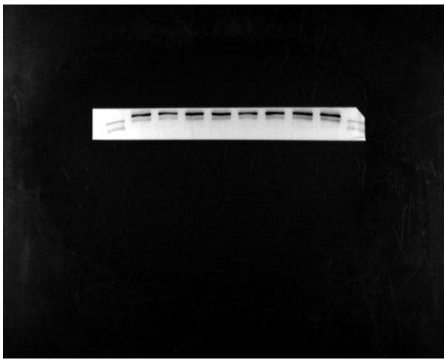

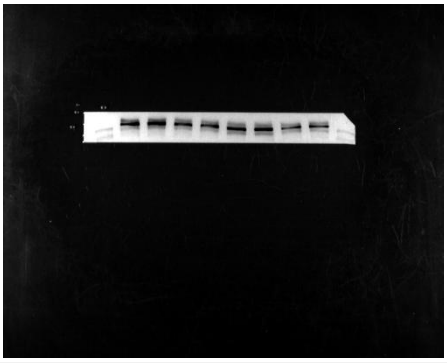

Hep

Probed with anti-mTOR antibody

Probed with Phosphorylation-mTOR antibody

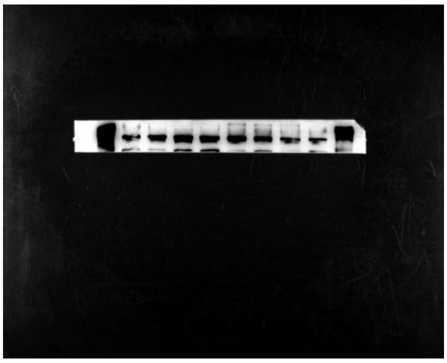

Probed with anti-PI3K antibody

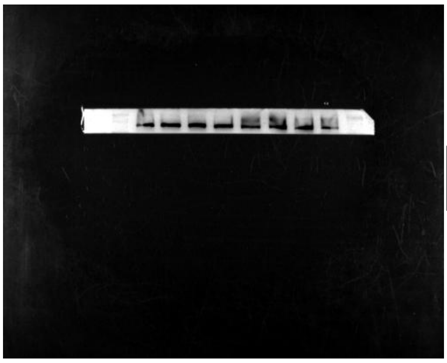

Hep G2

Probed with Phosphorylation-PI3K antibody

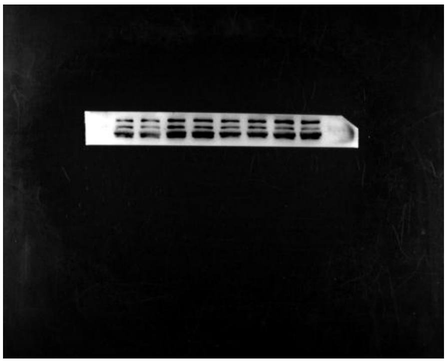

Probed with anti-Akt antibody

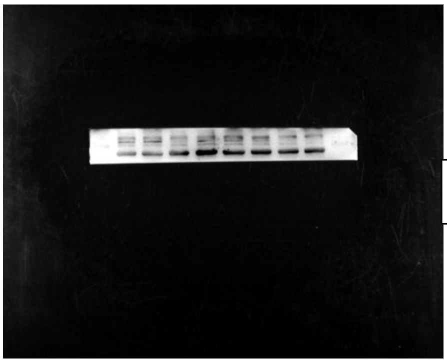

Hep G2

Probed with Phosphorylation-Akt antibody

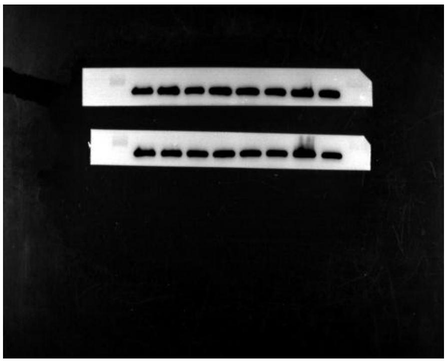

Hep G2

Probed with GAPDH antibody

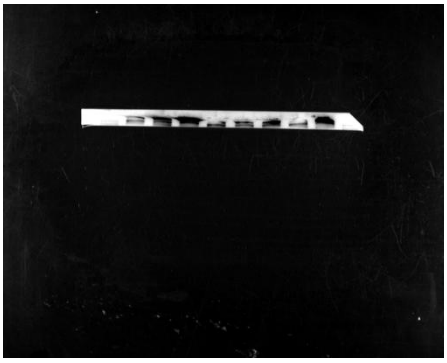

Probed with anti-mTOR antibody

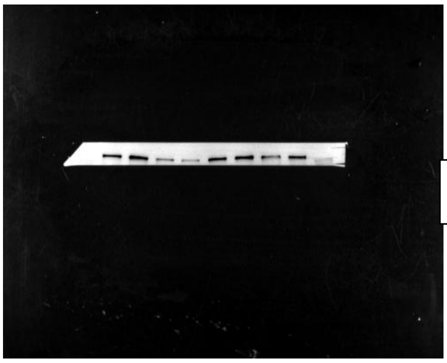

SMMC7721

Probed with Phosphorylation-mTOR antibody

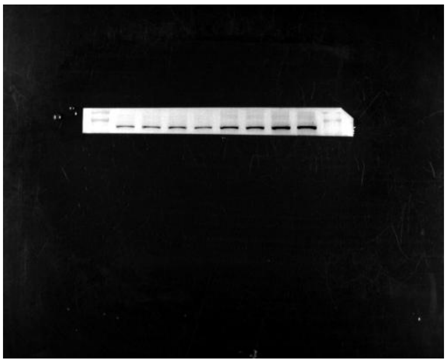

Probed with anti-PI3K antibody

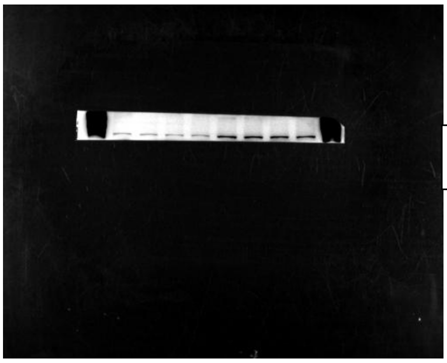

SMMC7721

Probed with Phosphorylation-PI3K antibody

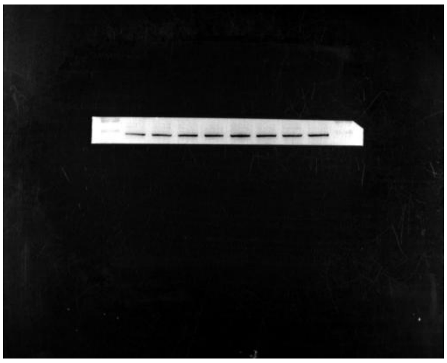

Probed with anti-Akt antibody

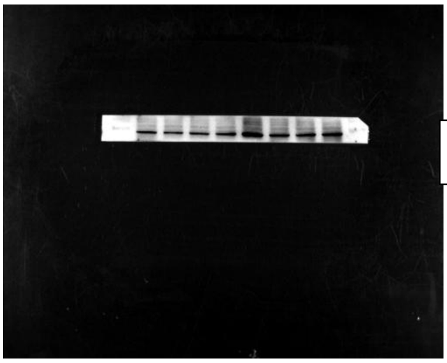

SMMC7721

Probed with Phosphorylation-Akt antibody

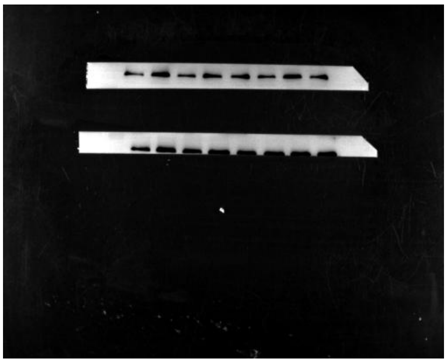

SMMC7721

Probed with GAPDH antibody

Supplementary Figure 6. ZNF207 affects the PI3K/Akt signaling pathway in Hep G2. (A) Western blot analysis of PI3K, Akt and mTOR in Hep G2. (B) The densiometric analysis for PI3K, Akt and mTOR in Hep G2. *ns*, no significance, a: $p < 0.05$, b: $p < 0.01$. (C) The original blots of Supplementary Figure S6A illustrate the following: the top membrane was stained with anti-mTOR & p-mTOR antibody (left) and anti-GAPDH antibody (right) in Hep G2, the middle membrane was probed with anti-PI3K antibody (left) and p-PI3K antibody (right) in Hep G2. The lower membrane was probed with anti-Akt antibody (left) and p-Akt antibody (right) in Hep G2. (D) The original blots of Figure 6A illustrate the following: the top membrane was stained with anti-mTOR & p-mTOR antibody (left) and anti-GAPDH antibody (right) in Hep G2, the middle membrane was probed with anti-PI3K antibody (left) and p-PI3K antibody (right) in Hep G2. The lower membrane was probed with anti-Akt antibody (left) and p-Akt antibody (right) in Hep G2. The rest membranes in

SMMC 7721 were probed with antibody in equal order.
