# Supplementary material for: Downregulation of hnRNPA1 inhibits hepatocellular carcinoma cell progression by modulating alternative splicing of ZNF207 exon 9
Source: Front Oncol. 2025 Jan 6;14:1517459. doi: 10.3389/fonc.2024.1517459 (PMC11743940; doi:10.3389/fonc.2024.1517459)
Supplement: Supplementary file 2 [file Table1.pdf]

Table S1 Genes Primers and siRNA sequence

Primers name	Sequences（5’→3’）
ACTIN-F	CGTGGACATCCGCAAAGACC
ACTIN-R	CGTCATACTCCTGCTTGCTG
GAPDH-F	GGTCTCCTCTGACTTCAACA
GAPDH-R	AGCCAAATTCGTTGTCATAC
BCL-2-F1	GGTGGGGTCATGTGTGTGG
BCL-2-R1	CGGTTCAGGTACTCAGTCATCC
hnRNPA1-qPCR-F1	TCAGAGTCTCCTAAAGAGCCC
hnRNPA1-qPCR-R1	ACCTTGTGTGGCCTTGCAT
CNND-qPCR-F1	CAATGACCCCGCACGATTTC
CNND-qPCR-R1	CATGGAGGGCGGATTGGAA
MPP2-qPCR-F1	CTTGCTCCAGATCGTAAACCAG
MPP2-qPCR-R1	CTCTGCATTCTTGGTGGTCAA
MMP9-qPCR-F1	GGGACGCAGACATCGTCATC
MMP9-qPCR-R1	TCGTCATCGTCGAAATGGGC
VEGFA-qPCR-F1	AGGGCAGAATCATCACGAAGT
VEGFA-qPCR-R1	AGGGTCTCGATTGGATGGCA
HIF1A-qPCR-F1	CACCACAGGACAGTACAGGAT
HIF1A-qPCR-R1	CGTGCTGAATAATACCACTCACA
BAX-qPCR-F1	CCCGAGAGGTCTTTTTCCGAG
BAX-qPCR-R1	CCAGCCCATGATGGTTCTGAT
siRNA-*hnRNPA1*	CAGCUGAGAGUAGACACUUGUGGUA
AS-delete-F	CCACAGCCTCCAGTTACTAAG
AS-delete-R	AGCAGGGAATGTAGGCTTTG
ZNF207-BalI-F	gcGTCGACcGGTCGCAAGAAGAAGAAGCA
ZNF207-KpnI-R	ggGGTACCtcagtaacggccaccttgcg
ZNF207-OE-F	tgctggacagGCTCAGGCAGCTGTCCAAGG
ZNF207-OE-R	ctgcctgagcCTGTCCAGCACTGGGGAAAAGAGGC
ZNF207-3’UTR-F	GGACCACTGCCTGAAAGGTT
ZNF207-3’UTR-R	ACCATAGAGGGTGGAAGGGT
